# The core autophagy machinery is not required for chloroplast singlet oxygen-mediated cell death in the *Arabidopsis thaliana plastid ferrochelatase two* mutant

**DOI:** 10.1186/s12870-021-03119-x

**Published:** 2021-07-19

**Authors:** Matthew D. Lemke, Karen E. Fisher, Marta A. Kozlowska, David W. Tano, Jesse D. Woodson

**Affiliations:** The School of Plant Sciences, University of Arizona, Tucson, AZ 85721-0036 USA

**Keywords:** Abiotic stress, Autophagy, Cellular degradation, Chloroplast, Microautophagy, Oxidative stress, Photosynthesis, Reactive oxygen species, Signaling, Singlet oxygen

## Abstract

**Background:**

Chloroplasts respond to stress and changes in the environment by producing reactive oxygen species (ROS) that have specific signaling abilities. The ROS singlet oxygen (^1^O_2_) is unique in that it can signal to initiate cellular degradation including the selective degradation of damaged chloroplasts. This chloroplast quality control pathway can be monitored in the *Arabidopsis* *thaliana* mutant *plastid ferrochelatase two* (*fc2*) that conditionally accumulates chloroplast ^1^O_2_ under diurnal light cycling conditions leading to rapid chloroplast degradation and eventual cell death. The cellular machinery involved in such degradation, however, remains unknown. Recently, it was demonstrated that whole damaged chloroplasts can be transported to the central vacuole via a process requiring autophagosomes and core components of the autophagy machinery. The relationship between this process, referred to as chlorophagy, and the degradation of ^1^O_2_-stressed chloroplasts and cells has remained unexplored.

**Results:**

To further understand ^1^O_2_-induced cellular degradation and determine what role autophagy may play, the expression of autophagy-related genes was monitored in ^1^O_2_-stressed *fc2* seedlings and found to be induced. Although autophagosomes were present in *fc2* cells, they did not associate with chloroplasts during ^1^O_2_ stress. Mutations affecting the core autophagy machinery (*atg5*, *atg7*, and *atg10*) were unable to suppress ^1^O_2_-induced cell death or chloroplast protrusion into the central vacuole, suggesting autophagosome formation is dispensable for such ^1^O_2_–mediated cellular degradation. However, both *atg5* and *atg7* led to specific defects in chloroplast ultrastructure and photosynthetic efficiencies, suggesting core autophagy machinery is involved in protecting chloroplasts from photo-oxidative damage. Finally, genes predicted to be involved in microautophagy were shown to be induced in stressed *fc2* seedlings, indicating a possible role for an alternate form of autophagy in the dismantling of ^1^O_2_-damaged chloroplasts.

**Conclusions:**

Our results support the hypothesis that ^1^O_2_-dependent cell death is independent from autophagosome formation, canonical autophagy, and chlorophagy. Furthermore, autophagosome-independent microautophagy may be involved in degrading ^1^O_2_-damaged chloroplasts. At the same time, canonical autophagy may still play a role in protecting chloroplasts from ^1^O_2_-induced photo-oxidative stress. Together, this suggests chloroplast function and degradation is a complex process utilizing multiple autophagy and degradation machineries, possibly depending on the type of stress or damage incurred.

**Supplementary Information:**

The online version contains supplementary material available at 10.1186/s12870-021-03119-x.

## Background

Plants have evolved intricate signaling systems that enable them to sense and subsequently respond to environmental stresses. One way plants achieve this is through the use of their chloroplasts (specialized photosynthetic plastid organelles) as sensors for multiple types of abiotic and biotic stresses. This is due, in part, to the unique photochemistry in chloroplasts that leads to the production of the reactive oxygen species (ROS) superoxide and singlet oxygen (^1^O_2_) at photosystems I (PSI) and II (PSII), respectively [1]. Such ROS production can occur even under ideal conditions and plants have evolved mechanisms to combat it, including non-photochemical quenching (NPQ), the production of ROS quenching pigments and enzymes, and systems to regulate energy capture and photosynthetic activity [2–4]. However, chloroplast ROS may further accumulate under various stresses including excess light (EL) [5], drought [6], salinity [7], and pathogen attack [8]. In such cases, these ROS may then overwhelm the protective mechanisms and lead to chloroplast and photosystem damage through the oxidation of proteins, DNA, and lipids [5]. At the same time, chloroplast ROS also act as signaling molecules [6, 9]. Both hydrogen peroxide (produced after the dismutation of superoxide) and ^1^O_2_ have been demonstrated to induce separate signals to control the expression of hundreds of nuclear genes [10]. Moreover, ^1^O_2_ has been shown to induce programmed cellular degradation, including selective chloroplast degradation [11, 12].

The ability of ^1^O_2_ to induce such cellular degradation has been inferred from multiple *Arabidopsis* *thaliana* mutants that specifically and conditionally accumulate large amounts of chloroplast ^1^O_2_ [12]. One such mutant, *plastid ferrochelatase two* (*fc2*), is defective in one of the two conserved chloroplast enzymes required for heme synthesis. Under diurnal light cycling conditions, this mutant accumulates the substrate of the FC2 enzyme, protoporphyrin IX (PPIX), an intermediate of the tetrapyrrole (e.g., chlorophylls and hemes) biosynthetic pathway [13]. Like other unbound tetrapyrroles, the accumulation of free PPIX leads to the rapid production of ^1^O_2_ in the light [14]. Within hours, such high ^1^O_2_ levels lead to wholesale chloroplast degradation followed by cell death. This can be easily visualized in seedlings or adult plants by the rapid bleaching of cotyledons and necrotic lesions in leaves, respectively [15, 16]. Even when *fc2* mutants are grown under permissive 24 h constant light conditions that avoid cell death, chloroplasts are still observed to degrade within the cytoplasm. These degrading chloroplasts can sometimes be observed protruding or blebbing into the central vacuole of an otherwise healthy cell. Together, it was hypothesized that certain chloroplasts are selectively degraded in this mutant [15, 16]. However, the relationship between cell death and chloroplast degradation is unknown. Cell death may be a consequence of excess chloroplast degradation or an independent signal from severely damaged chloroplasts. In either case, the mechanisms controlling this degradation and the subsequent transport of chloroplast material into the central vacuole are also unknown.

^1^O_2_ is naturally produced at PSII during EL and other environmental stresses that inhibit photosynthesis [5, 17]. Under such conditions, chlorophylls in PSII become unable to transfer their energy to the photosynthetic reaction centers, excite to their triplet state, and interact with molecular oxygen to generate ^1^O_2_. Unlike hydrogen peroxide that is simultaneously made during most of these stresses, ^1^O_2_ has an extremely short half-life (0.5–1 μsec in cells [18]) and the bulk of ^1^O_2_ is likely confined to the same chloroplast in which it was generated (^1^O_2_ can travel < 200 nm, but chloroplasts are 5–10 μm wide). A portion of ^1^O_2_ may exit the chloroplast (^1^O_2_ has been detected in the apoplast of light-stressed Arabidopsis cells [19]), but due to its high reactivity it is expected that secondary signals controlling nuclear gene expression and/or chloroplast degradation in response to ^1^O_2_ accumulation likely exist [15, 20]. To identify such signaling factors involved in ^1^O_2_-induced chloroplast degradation, a genetic screen for *ferrochelatase two suppressor* (*fts*) mutations that block cell death and chloroplast degradation in the *fc2* mutant have been performed [15]. Three *fts* mutants have been shown to affect *PENTATRICOPEPTIDE-REPEAT-CONTAINING PROTEIN 30* (*PPR30*) and “*mitochondrial*” *TRANSCRIPTION TERMINATION FACTOR 9* (*mTERF9*) [16]. Both genes encode chloroplast-localized proteins predicted to be involved in post-transcriptional gene regulation; PPR30 belongs to the P-class of PPR proteins that regulate gene expression by directly affecting RNA transcript stability, processing, editing, and/or translation [21] and mTERF9 has been demonstrated to be required for assembly of the 30S ribosome subunit in chloroplasts [22]. As such, these results suggest a (currently unidentified) chloroplast genome-encoded factor(s) may be required to initiate the ^1^O_2_ signal.

A fourth *fts* mutant was shown to affect the cytoplasmic ubiquitin E3 ligase Plant U-Box 4 (PUB4) suggesting the cellular ubiquitination machinery is involved in targeting damaged chloroplasts for degradation [15]. Consistent with this hypothesis, proteins associated with ^1^O_2_-stressed chloroplasts became ubiquitinated prior to degradation. This degradation was mostly blocked in the *fc2 pub4-6* double mutant, indicating PUB4, or another E3 ligase marks damaged chloroplasts for turnover by ubiquitination [15]. As such, chloroplast generated ^1^O_2_ may induce a chloroplast quality control system allowing cells to sense and turnover damaged chloroplasts to sustain healthy chloroplast populations and maintain efficient energy production. Which cellular machinery is involved in the targeted degradation and removal of these chloroplasts, however, remains unknown. Interestingly, the *pub4-6* mutation also blocks selective chloroplast degradation under permissive constant light conditions (in both wt and *fc2* backgrounds). This suggests (at least some) *fts* mutations block ^1^O_2_-induced cell death by reducing chloroplast degradation [15]. Alternatively, chloroplast ^1^O_2_ signals may induce chloroplast degradation and cell death separately, each involving different cellular degradation machineries.

Cellular quality control systems are fundamental mechanisms in biology that allow organisms to maintain the optimal functionality of molecular processes. In the case of damaged proteins, cellular components, or even whole organelles, autophagy often plays a role in such processes [23, 24]. A conserved process in eukaryotes, autophagy allows cells to degrade and recycle macromolecules and larger cellular components such as whole organelles [25, 26]. In mammals, ROS-induced mitochondrial degradation (mitophagy) is regulated by the E3 ubiquitin ligase PARKIN, leading to the autophagic removal of mitochondria to the lysosome [27].

In eukaryotic cells, two major types of autophagy are used; macroautophagy and microautophagy. Macroautophagy is a well-studied and fundamental process that is strongly conserved across eukaryotic kingdoms [28]. In plants, canonical macroautophagy is generally characterized as a process whereby cytosolic components are encapsulated in a double membrane vesicle, known as an autophagosome, and are then transported to the central vacuole for turnover and eventual remobilization of nutrients [29, 30]. This process is fundamental in the maintenance of cellular homeostasis, nutrient storage, and degradation of cytotoxic components and dysfunctional proteins [31, 32]. This form of autophagy has been well characterized and requires core autophagy (ATG) proteins such as Autophagy 5 (ATG5) and Autophagy 7 (ATG7). Such proteins play key roles in the Autophagy 8 (ATG8) conjugation system, a process required for the formation and maturation of autophagosomes, which encapsulate and package cytosolic components [33]. Alternatively, cytosolic components can be removed by invagination of the vacuole in a processes resembling endocytosis via microautophagy [34, 35]. Compared to macroautophagy, significantly less is known about microautophagy, particularly in plants [34]. However, despite the name, microautophagy processes are able to degrade almost any cellular component, including organelles. Also, as currently defined, microautophagy likely represents at least two types of cellular degradation processes; one dependent on the core autophagy proteins and autophagosome formation (ATG-dependent) and one independent of these proteins and structures (ATG-independent) [36].

Recent work has suggested whole chloroplasts are also transported to the central vacuole for degradation, either by processes that resemble macroautophagy or microautophagy, after damage by UVB [37] or excess light (EL) [38], respectively. Under UVB stress, damaged chloroplasts are completely engulfed by autophagosomes, a hallmark of macroautophagy. Under EL stress, however, only partial autophagosomes associate with damaged chloroplasts. This process resembles ATG-dependent microautophagy, where cytoplasmic components are transported into the central vacuole with only partial contact with an autophagosome [38], and is reminiscent of the ATG-dependent microautophagic degradation of peroxisome organelles (micropexophagy) in yeast [36]. The specific mechanisms involved in these processes (collectively dubbed chlorophagy) remain unclear, but it is evident that both are dependent on core autophagy proteins. Under both UVB and EL stress, the transport of damaged chloroplasts to the central vacuole is blocked in *atg5* and *atg7* mutants, suggesting autophagosomes or phagophores are required to proceed and complete chlorophagy [38, 39]. Recent work has shown that PUB4 is dispensable to chlorophagy during EL stress, suggesting that this E3 ligase is not necessary for autophagosome formation or chlorophagy [40]. However, the relationship between autophagy and chloroplast quality control during ^1^O_2_ stress has remained unexplored.

In this study, we sought to further understand the mechanisms involved in ^1^O_2_-induced chloroplast quality control and cell death in *fc2* mutants by determining what role autophagy may play in these pathways. Although a large number of autophagy-related genes are induced in stressed *fc2* seedlings, autophagosomes do not interact with these ^1^O_2_-stressed chloroplasts. Moreover, mutations affecting the core autophagy machinery do not fully block chloroplast turnover or cell death in the *fc2* mutant. Finally, our gene expression analyses point towards a potential role for ATG-independent microautophagy in ^1^O_2_-induced chloroplast quality control. Together, these results suggest that multiple chloroplast degradation pathways likely exist in plant systems and that chloroplast quality control itself is a complex process regulated by multiple types of cellular autophagy machinery.

## Results

### Autophagy is transcriptionally induced in stressed *fc2* mutant seedlings

In *fc2* mutants, the accumulation of ^1^O_2_ in chloroplasts leads to their selective degradation and eventual cell death [15]. In such cases, degrading chloroplasts appear to interact with the central vacuole where they may be further degraded and recycled. While UVB and EL damaged chloroplasts have been shown to be transported to the vacuole in an autophagy-dependent manner [38, 39], the cellular machinery involved in transporting ^1^O_2_-damaged chloroplasts in the *fc2-1* mutant is unknown.

**Fig. 1:**
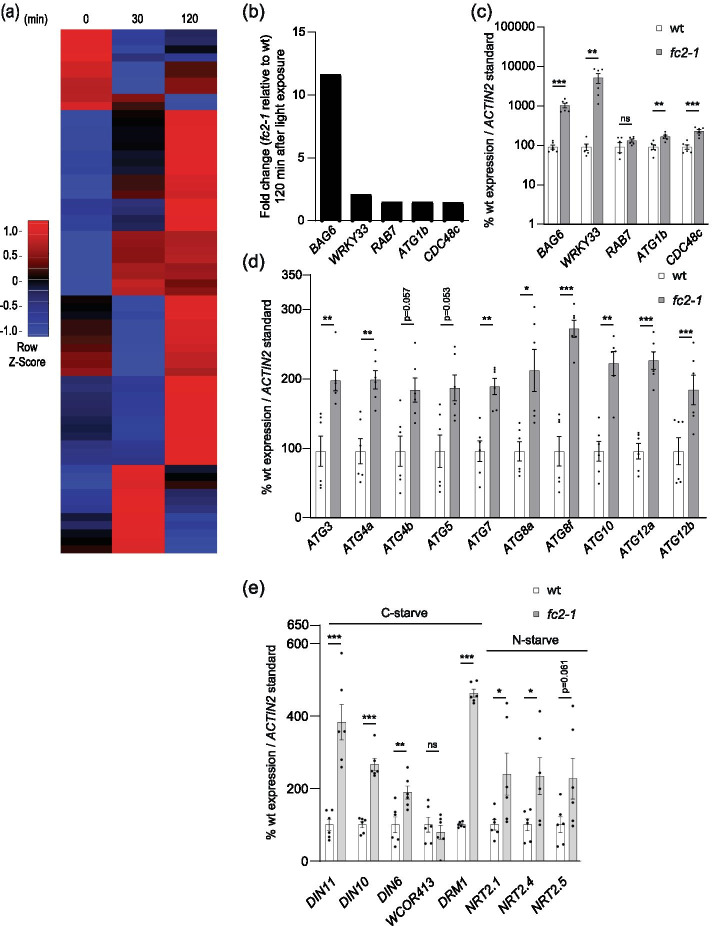
Autophagy-related genes are transcriptionally induced in stressed *fc2-1* seedlings. The expression of autophagy-related genes was monitored in *fc2-1* mutants. **A** A heatmap of autophagy-related gene expression (relative to wt) in etiolated seedlings at time points prior to (0 min) and during de-etiolation (30 min and 120 min). Microarray data was previously generated from etiolated seedlings grown in the dark for four days and exposed to light for the indicated amount of time [15]. The list of genes considered is presented in Table S1. **B** Expression fold change (relative to wt) of the top five most up-regulated core autophagy and autophagy genes at 120 min from panel A. **C**, **D**, and **E** RT-qPCR analysis of autophagy-related, core autophagy, and starvation (carbon and nitrogen) marker transcripts, respectively, from four-day-old seedlings grown under 6 h light/18 h dark light cycling conditions. Shown are mean values ± SEM (n = 6 biological replicates). Statistical analyses were performed by student’s t-tests. *, **, *** indicate a p-value of ≤ 0.05, ≤ 0.01, and ≤ 0.001, respectively. In all bar graphs, closed circles represent individual data points

To investigate if autophagy may also be involved in degrading chloroplasts and/or cell death in *fc2* mutants, we first tested if core autophagy and autophagy-related genes are transcriptionally induced by ^1^O_2_ accumulation. To do this, we turned to a previously published microarray data set of etiolated (dark-grown) wild type (wt) and *fc2-1* seedlings before and immediately after light exposure (30 and 120 min) [15]. Under such conditions where plants are shifted from dark to light, *fc2* mutants accumulate ^1^O_2_ and initiate cellular degradation. We then analyzed the expression of a manually curated list of 71 (65 included in the microarray data set) core autophagy and autophagy-related genes (Table S1). At 120 min, 13 out of 65 genes (20%) were significantly induced in *fc2-1* compared to wt (fold change ≥ 1.25, false discovery rate (FDR) ≤ 0.05). A heatmap analysis showed a general (yet mild) pattern of induction of these autophagy-related genes across both time points (Fig. 1a). At 120 min, the top five most highly induced autophagy-related genes were *BAG6* (*AT2G46240*), *WRKY33* (*AT2G38470*), *RAB7* (AT1G22740), *ATG1B* (*AT3G53930*), and *CDC48C* (*AT5G03340*) (Fig. 1b). Of particular interest is *BAG6*, a strongly conserved positive regulator of programmed cell death [41] and the most strongly induced differentially expressed gene (at 120 min) from our manually curated list. To determine if *fc2-1* seedlings respond similarly under light cycling conditions, we monitored the expression of these five genes in four-day old seedlings grown in 6 h light/18 h dark cycling light conditions. Under such cycling light conditions, ^1^O_2_ accumulates in the chloroplasts of *fc2-1* mutants and leads to chloroplast degradation and cell death [15, 16]. Compared to wt, four of the five genes (excepting *RAB7*) were significantly induced in *fc2-1* seedlings suggesting these expression patterns are maintained post de-etiolation (Fig. 1c).

As autophagy-related genes appear to have a general induction pattern in stressed *fc2-1* seedlings, we probed ten core autophagy genes that make up the ATG8-conjugation system to explore if macroautophagy or autophagosome formation may be transcriptionally induced. Again, we saw a similar trend in the induction of these genes under 6 h light/18 h dark cycling light conditions. All ten genes (eight significantly, p values ≤ 0.05) were induced in *fc2-1* compared to wt (Fig. 1d). Together, these data demonstrate that a subset of autophagy-related genes, including those that compose the ATG8-conjugation system, are transcriptionally induced in ^1^O_2_-stressed *fc2-1* seedlings during and after de-etiolation and suggest macroautophagy is activated in response to chloroplast ^1^O_2_ stress.

As photosynthesis is likely impaired in *fc2-1* seedlings [13, 15] and as carbon (C) and nitrogen (N) starvation have been shown to play a role in the induction of autophagy [42], we explored the possibility *fc2-1* seedlings are exhibiting a starvation response. To this end, we probed a selection of C- and N-starvation marker genes [42] in four day old seedlings grown under 6 h light/18 h dark cycling light conditions. Compared to wt, the C-starvation marker genes (*DIN11* (*AT3G49620*), *DIN10* (*AT5G20250*) [43], *DIN6* (*AT3G47340*), and *DRM1* (*AT1G28330*) [44]) were significantly induced in *fc2-1* seedlings (Fig. 1e). The expression of one marker gene, *WCOR413* (*AT4G37220*), was not significantly different between wt and *fc2-1*. To investigate an N-starvation response, we monitored three markers genes; *AT1G08090* (*NRT2.1*), *AT5G60770* (*NRT2.4*), *AT1G12940* (*NRT2.5*) [45]. Two of these genes, *NRT2.1* and *NRT2.4*, were significantly induced in *fc2-1* compared to wt (Fig. 1e). Together, these results suggest that stressed *fc2-1* seedlings are experiencing C- and N-starvation responses that may contribute to the transcriptional induction of autophagy-related genes.

### ATG8 does not associate with stressed chloroplasts in *fc2* mutants

As autophagy-related genes are transcriptionally induced in ^1^O_2_-stressed *fc2-1* mutants, we next sought to determine if canonical macroautophagy itself is active in *fc2-1* seedlings and if autophagosomes associate with ^1^O_2_-stressed chloroplasts. Such a response can be monitored by following the localization of the ATG8 protein, a post-transcriptionally regulated hallmark of autophagosome formation, macroautophagy, and ATG-dependent microautophagy [46]. In Arabidopsis, GFP fused ATG8 proteins have been observed to form punctae and circular structures upon induction of autophagy [47]. During chlorophagy, for instance, GFP-ATG8a can be observed as tubular formations interacting with chloroplasts after UVB or EL stress [37, 39].

**Fig. 2:**
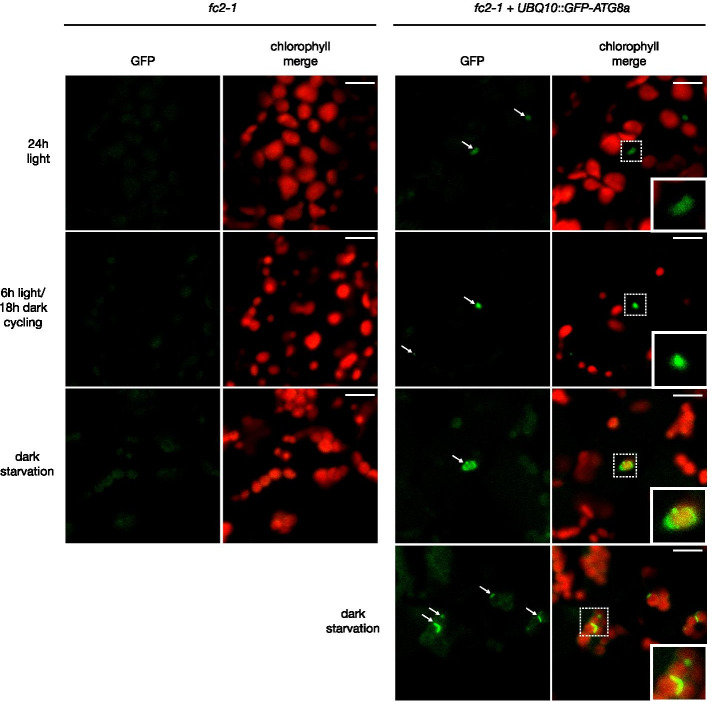
ATG8a does not associate with ^1^O_2_-stressed chloroplasts in *fc2* seedlings. The localization of GFP-ATG8a was assessed in stressed *fc2-1* seedlings. Shown are representative images of GFP-ATG8a in four day-old *fc2-1* seedlings grown in 24 h constant light or 6 h light/18 h dark cycling light. The same line was also subjected to dark-induced carbon starvation for six additional days. Also shown are representative images of untransformed *fc2-1* plants from each condition. Shown are the GFP signal (green) and merged overlays with chlorophyll autofluorescence (red). White arrows indicate GFP-ATG8a punctae in cells. Boxes with dashed lines mark magnified sections included on bottom right of select panels. Scale bars = 10 µm

To determine if ATG8 and autophagosomes also interact with ^1^O_2_ stressed chloroplasts in the *fc2-1* mutant, we introduced a *UBQ10*::*GFP-ATG8a* construct into the *fc2-1* background and monitored GFP structures using confocal microscopy. Under both 24 h constant and 6 h light/18 h dark cycling light conditions, GFP punctae were observed in *fc2* cells, but did not appear to interact with chloroplasts under either condition (Fig. 2). A plot profile analysis of GFP and chlorophyll fluorescence confirmed this spatial separation (Fig. S1ab). To determine if the GFP-ATG8a fusion protein could sometimes interact with chloroplasts in the *fc2-1* mutant, we monitored GFP in dark-starved conditions (four days of light and six days of dark). Similar treatments have been shown to induce autophagy-dependent turnover of chloroplasts [48] and GFP-ATG8a has been shown to associate with chloroplasts during autophagy [49]. Indeed, under these starvation conditions, GFP-ATG8a did associate closely with chloroplasts, confirming that it is a marker for autophagy-dependent chloroplast degradation (Figs. 2 and S1ab). Together, these results suggest autophagosomes are present in *fc2-1* seedling mesophyll cells, but are not associating directly with ^1^O_2_-stressed chloroplasts.

### Blocking ATG-dependent autophagy does not suppress cell death in *fc2* seedlings

The above results suggest autophagosomes are active in *fc2* cells, but do not directly target ^1^O_2_-stressed chloroplasts for degradation. However, it is also possible that such interactions are too transient to detect, or that only a small portion of autophagosomes is involved in chloroplast stress. Therefore, to further investigate the role of ATG-dependent autophagy in ^1^O_2_-mediated chloroplast degradation and cell death, we tested if there is a genetic interaction between chloroplast quality control and the core autophagy machinery in *fc2* seedlings. In *Arabidopsis*, core autophagy (ATG) proteins such as ATG5, ATG7, and Autophagy 10 (ATG10), have been shown to be essential for autophagosome formation and thus are required for canonical macroautophagy [50–52]. In addition, both ATG5 and ATG7 have been shown to be specifically required for chlorophagy [38, 39]. Therefore, we introduced null *atg5-1*, *atg7-2*, and *atg10-1* mutations into the *fc2-1* background (confirmed via PCR genotyping (Fig S2a)). An RT-qPCR analysis confirmed the three *fc2-1 atg* double mutants lacked their respective *ATG* transcripts (Fig. S2b) and exhibited the expected early senescence phenotypes caused by these mutations (Fig. S2c) [50, 52, 53].

**Fig. 3:**
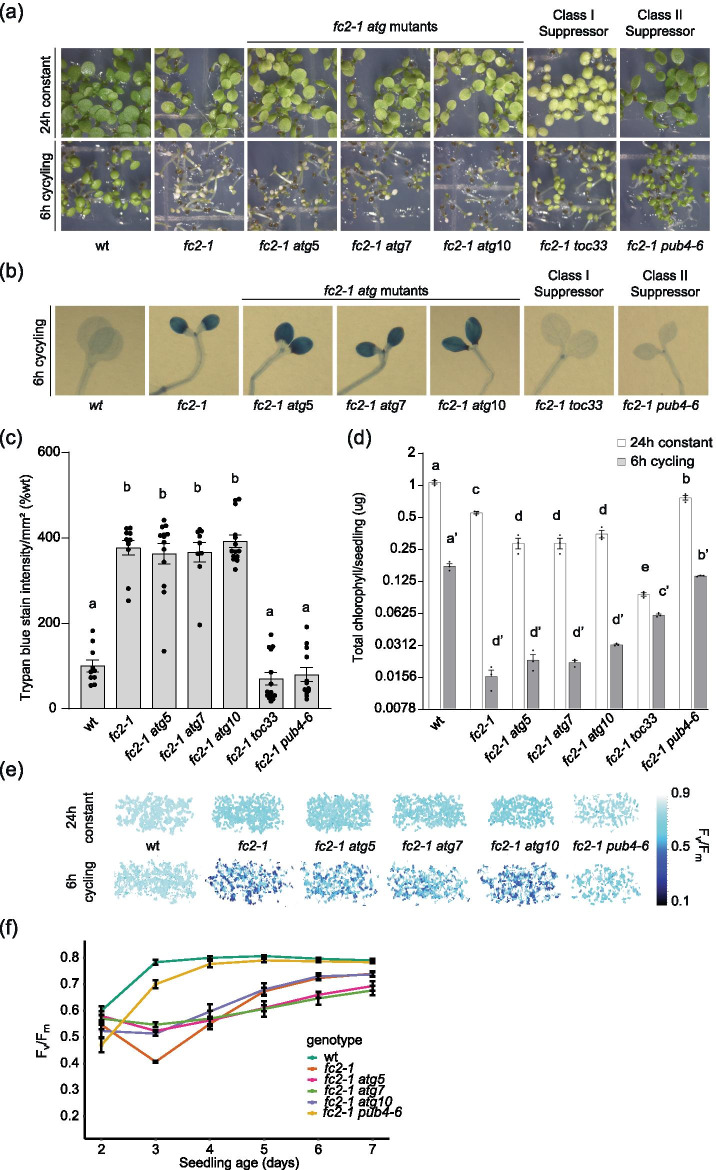
Blocking ATG-dependent autophagy does not suppress ^1^O_2_-induced cell death in *fc2* seedlings. *atg* mutations were tested for their ability to suppress cell death in the *fc2-1* mutant. **A** Seven-day old seedlings grown in constant light (24 h) or 6 h light/18 h dark (6 h) cycling light conditions. **B** Trypan blue stains of seedlings from panel A. The dark blue color is indicative of cell death. **C** Mean values (± SEM) of the trypan blue signal in panel B (n ≥ 10 seedlings). **D** Mean chlorophyll content (µg/seedling) (± SEM) of groups of six-day old seedlings grown in 24 h light or 6 h cycling light conditions (n = 3 biological replicates)(y-axis in log_2_ scale). **E** Representative images of maximum quantum yield of PSII (F_v_/F_m_) measured from three-day-old seedlings grown in the indicated light regiment. **F** Changes in F_v_/F_m_ of seedlings grown over seven days in 6 h cycling light conditions. Mean values are shown ± SEM (n = 3 biological replicates). Statistical analyses were performed by one-way ANOVA tests followed by Tukey's HSD. Different letters above bars indicate significant differences (p value ≤ 0.05). For panel D, separate statistical analyses were performed for the different light treatments and the significance for the 6 h cycling values is denoted by letters with a ʹ. In all bar graphs, closed circles represent individual data points

Although the connection between chloroplast degradation and cell death in *fc2* mutants is not well understood, these phenotypes are genetically linked (suppressors of cell death also suppress chloroplast degradation). Therefore, if autophagy is required for ^1^O_2_-induced chloroplast degradation, it may also reduce cell death in *fc2* mutants under cycling light conditions [15, 16]. As such, we next tested the ability of these *atg* mutations to suppress the *fc2-1* cell death phenotype, which can be achieved by reducing ^1^O_2_ accumulation (as is known for class I mutants such as *toc33*/*fts4*, which is defective in a component of the outer envelope plastid protein import machinery) or by blocking the ^1^O_2_ signal (as is known for class II mutants such as *pub4-6*/*fts29*) [15]. As expected under 6 h light/18 h dark cycling light conditions, *fc2-1* mutants suffer from cell death and fail to green (Fig. 3a). This was suppressed by *toc33* and *pub4-6,* but not by any of the three *atg* mutations. This cell death was dependent on light cycling as these mutants appeared healthy under permissive 24 h constant light conditions. Furthermore, in the wt background, all three *atg* single mutants appeared healthy suggesting that cell death was dependent on the *fc2-1* background (Fig. S3a). Cell death itself was confirmed through trypan blue staining of the seedlings (Figs. 3b and c and S2b and c) and there was no significant difference between *fc2-1* and the *fc2-1 atg* double mutants under light cycling conditions.

Additionally, the low chlorophyll content of *fc2-1* seedlings grown under cycling light is not significantly affected by the three *atg* mutations, further demonstrating their inability to suppress the *fc2-1* cell death phenotype (Fig. 3d). Compared to *fc2-1*, however, *fc2-1 atg* double mutants have a significantly lower level of chlorophyll in 24 h constant light conditions, which may indicate a role for autophagy in chloroplast development or stress responses. This effect on chlorophyll content is partially independent in the *fc2-1* background, as single *atg* mutants have reduced chlorophyll content compared to wt (Fig. S3d). Together, these data demonstrate *atg* mutations do not suppress the *fc2-1* cell death phenotype, indicating core autophagy proteins ATG5, ATG7, and ATG10 are dispensable in ^1^O_2_-mediated cell death at the seedling stage.

To further investigate the effect of *atg* mutations on the physiology of *fc2-1* seedlings, we next measured maximum quantum yield of PSII (F_v_/F_m_), an indicator of photoinhibition and plant stress [54], in the same seedlings. Under 24 h constant light conditions, photosynthetic efficiency is slightly decreased in *fc2-1* mutants compared to wt, but gradually increases to wt levels after five days (Fig. 3e and S3a). Under 6 h light/18 h dark cycling light conditions, however, photosynthetic efficiency in *fc2-1* mutants decreases at day three and then gradually recovers over the next four days (Fig. 3e and f). This likely reflects the level of cellular degradation occurring in these mutants.

While the cell death and chloroplast degradation suppressor *pub4-6* mostly restored photosynthetic efficiency to the *fc2-1* mutant under cycling light conditions, the three *atg* mutations did not (Fig. 3e). However, a closer look at the time course analysis shows that the three *fc2-1 atg* double mutants are slightly less affected by ^1^O_2_ stress at day three compared to *fc2-1* (Fig. 3f), but then recover more slowly than *fc2-1* over the following four days. This effect is specific to cycling light as *fc2-1 atg* double mutants are nearly identical to *fc2-1* in 24 h constant light (Fig. S4a). Moreover, this effect is specific to the *fc2-1* background, as single *atg* mutants are almost indistinguishable from wt in either condition (Fig. S4b-d). Together, these results further support core autophagy machinery and ATG-dependent autophagy does not play a positive role in ^1^O_2_–induced cell death in the *fc2-1* mutant. However, the complex effect of the *atg* mutations on photosynthetic efficiency during ^1^O_2_ stress, suggests ATG-dependent autophagy may play a role in maintaining photosynthetic efficiency under photo-oxidative stress.

### Blocking ATG-dependent autophagy does not suppress cell death or growth inhibition in *fc2* adult plants

**Fig. 4:**
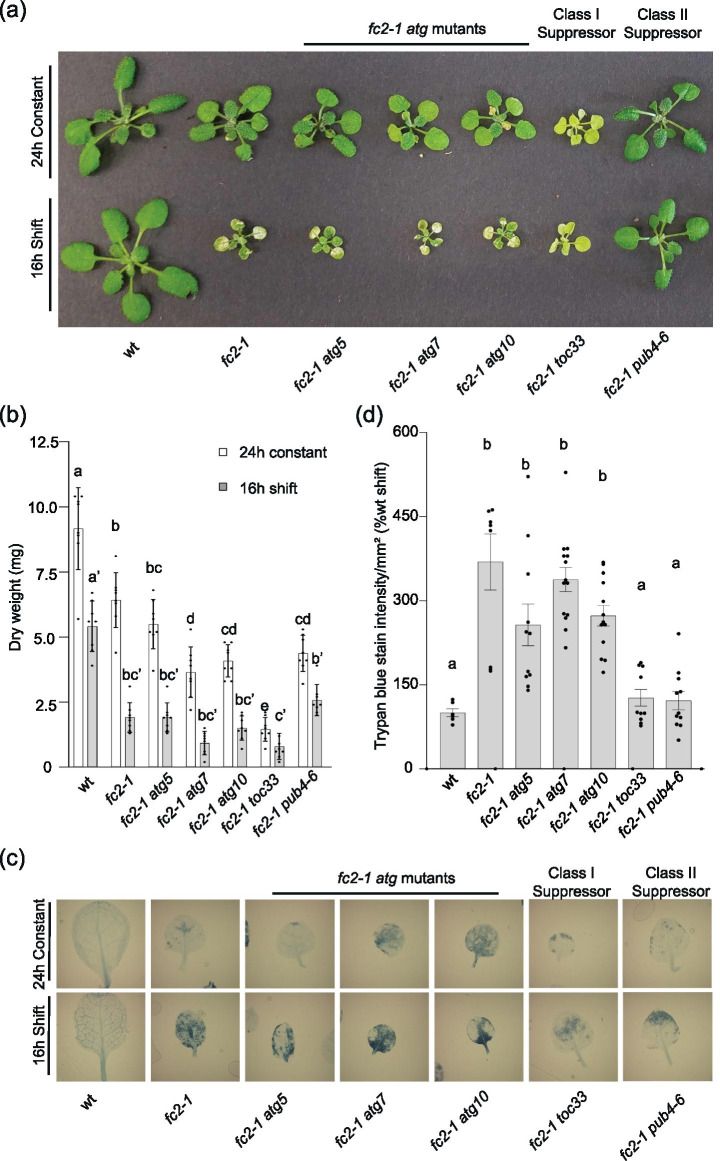
Blocking ATG-dependent autophagy does not suppress ^1^O_2_-induced cell death in *fc2* adult plants. The phenotypes of *fc2-1 atg* double mutant plants were assessed in the adult stage. **A** Three-week old plants grown in 24 h constant light or under stressed conditions (two weeks in 24 h constant light and one week in 16 h light/8 h dark cycling light conditions). **B** Mean biomass (± SD) of same plants (n = 8 plants). **C** Representative trypan blue stains of single leaves from same plants. Dark blue color is indicative of cell death. **D** Quantification of mean trypan blue signal (± SEM) from plants in 16 h light/8 h dark cycling light conditions (n ≥ 6 leaves from individual plants). Statistical analyses were performed by one-way ANOVA tests followed by Tukey's HSD. Different letters above bars indicate significant differences (p value ≤ 0.05). For panel B, separate statistical analyses were performed for the different light treatments and the significance for the light stressed group is denoted by letters with a ʹ. In all bar graphs, closed circles represent individual data points

Chlorophagy has previously been shown to occur in adult plant leaves after UVB and EL stress [38, 39]. Therefore, we tested if core autophagy machinery may play a stage-specific role in plant development by assessing the phenotypes of *fc2-1 atg* double mutant adult plants. To do this, we grew plants for two weeks in 24 h constant light and then transferred them to 16 h light/8 h dark cycling light conditions. As a control, another set of plants remained under 24 h constant light for a third week. While *fc2-1* plants are smaller than wt, but healthy even after three weeks in 24 h constant light, they exhibit necrotic lesions in leaves and strongly reduced growth (dry weight biomass) after one week in cycling light (Fig. 4a and b). These necrotic lesions were confirmed to be areas of cell death by trypan blue staining of leaves (Fig. 4c and d). When mature two-week-old *fc2-1 atg* double mutants were exposed to cycling light stress, all three had a similar amount of leaf necrosis, cell death, and impaired growth compared to *fc2-1* single mutants. The three single *atg* mutants behaved similarly to wt indicating the stress phenotypes described above are dependent on the *fc2-1* background (Fig. S5a-d). Together, these data suggest ^1^O_2_-induced cell death is not dependent on core autophagy machinery in either the seedling or adult stages.

### ATG-dependent autophagy is not required for transmitting the ^1^O_2_ retrograde signal in *fc2* mutants

In addition to chloroplast degradation and cell death, ^1^O_2_ also leads to retrograde signals that regulate hundreds of genes in the nucleus [11]. Under cycling light, stressed *fc2* mutants have been shown to have a clear induction of multiple nuclear stress marker genes (specific ^1^O_2_ response genes; *BAP1* (*AT3G61190*) and *ATPase* (*AT3G28580*) [10], general ROS response genes; *ZAT12* (*AT5G59820*) and *CYC81D8* (*At4g37370*) [55], and genes specific to stressed *fc2-1* seedlings; *SIB1* (*AT3G56710*) and *HSP26.5* (*AT1G52560*) [15]).

**Fig. 5:**
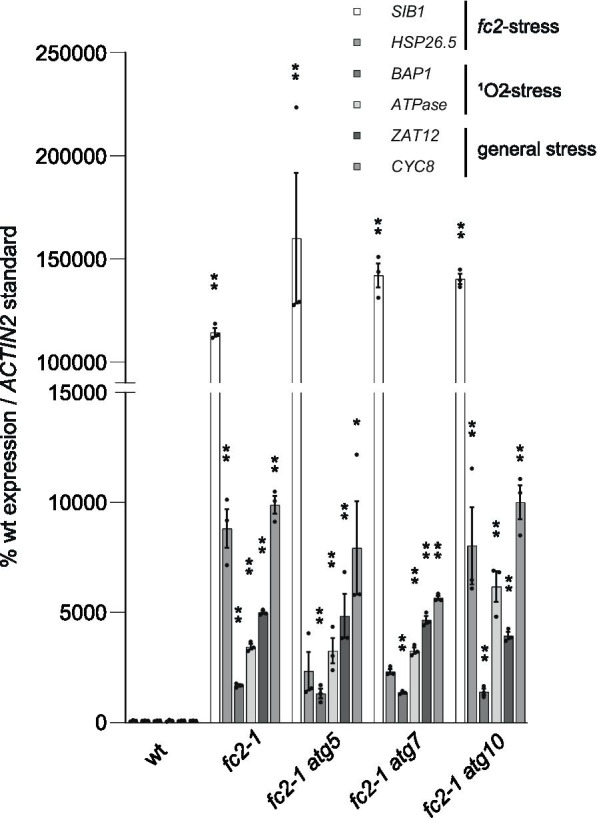
ATG-dependent autophagy is not required for ^1^O_2_ retrograde signaling in *fc2* mutants. The expression of nuclear genes controlled by chloroplast ^1^O_2_ stress signaling were monitored in the *fc2-1 atg* double mutants. RT-qPCR analyses of stress gene transcript abundance in four-day old *fc2-1* and *fc2-1 atg* double mutants grown under 6 h light/18 h dark cycling light conditions collected one hour after subjective dawn. Shown are means of biological replicates (n = 3) ± SEM. Statistical analyses were performed by a one-way ANOVA followed by Dunnett’s multiple comparisons test with the wt. * and ** indicates an adjusted p value of ≤ 0.05 and ≤ 0.01, respectively. Closed circles represent individual data points

As there is no apparent suppression of the physiological phenotypes of stressed *fc2-1* in the *fc2-1 atg* double mutants, we next asked if mutations in core autophagy genes lead to changes in the induction of these key stress markers. To do this, we extracted RNA from four-day-old seedlings grown under 6 h light/18 h dark cycling light conditions and used RT-qPCR to monitor transcript levels of these six marker genes. As expected, the *fc2-1* mutant significantly induced all six genes compared to wt (Fig. 5). The three *fc2-1 atg* double mutants also induced all six genes compared to wt and were virtually indistinguishable from the single *fc2-1* mutant. Together, these data suggest that core autophagy proteins are not necessary for the chloroplast retrograde signals induced in *fc2-1* seedlings.

### Chloroplast degradation in the *fc2-1* mutant is not dependent on ATG-dependent autophagy

The above results suggest the *atg* mutations do not lead to *fts* phenotypes and cannot block cellular degradation in the *fc2-1* mutant background under light cycling conditions. To test if the core autophagy machinery and macroautophagy instead play a specific role in dismantling ^1^O_2_-damaged chloroplasts in *fc2-1*, we used transmission electron microscopy to image four day old seedlings grown under permissive 24 h constant light conditions. Under such conditions, *fc2-1* mutants avoid cell death, but a subset of chloroplasts are still degraded and can sometimes be observed protruding or blebbing into the central vacuole [15].

**Fig. 6:**
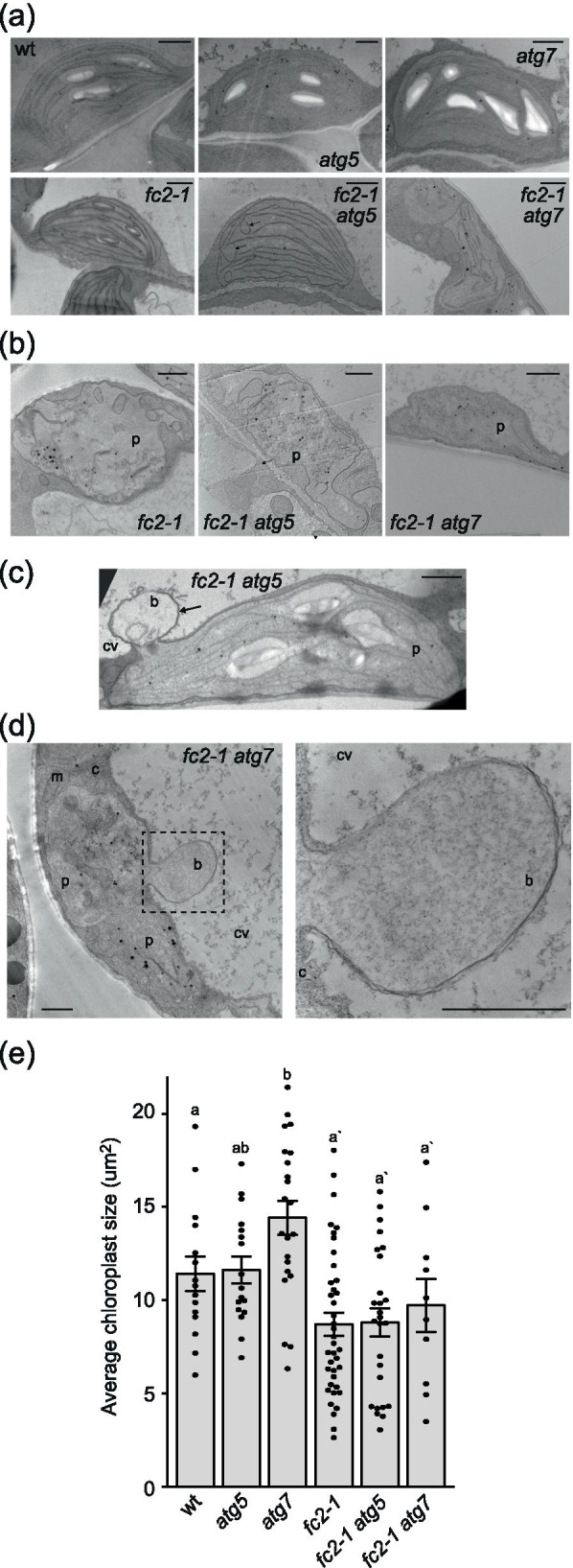
ATG-dependent autophagy is not required for selective chloroplast degradation in *fc2* mutants. Chloroplast ultrastructure was assessed by transmission electron microscopy (TEM) in the *atg* mutants. Shown are representative **A** intact and **B** degrading chloroplasts in four day old seedlings grown under 24 h constant light conditions. Arrows indicate unusual horseshoe membrane structures. Shown is a blebbing chloroplast interacting with the central vacuole in the **C** *fc2-1 atg5* and **D** *fc2-1 atg7* mutants. Arrows indicate the bleb-like structure protruding into the central vacuole. The right panel is a zoomed-in image of the boxed region. b; bleb, c; cytoplasm, cv; central vacuole, m; mitochondria, p; plastid. Bars = 1 µm. **E** Mean chloroplast area (± SEM) from the same seedlings (n ≥ 10). Statistical analyses were performed using one-way ANOVA tests followed by Tukey's HSD. Different letters above bars indicate significant differences (p value ≤ 0.05). Separate statistical analyses were performed for seedlings in the wt and *fc2-1* backgrounds and the significance for the *fc2-1* background group is denoted by letters with a ʹ. Closed circles represent individual data points

As expected, most of the chloroplasts in *fc2-1* seedlings appeared intact, but a subset was in the process of being degraded (Fig. 6a and b, Table 1) (18.6% of the total chloroplasts imaged). These degrading chloroplasts can be identified by their abnormal shapes, severely disorganized internal membranes, and compressed thylakoids. Most chloroplasts in *fc2-1 atg5* and *fc2-1 atg7* mutants also appeared intact, but a larger percentage were being degraded (39.0% and 45.3% in *fc2-1 atg5* and *fc2-1 atg7*, respectively) (Fig. 6a and b, Table 1). In some cases, chloroplasts in these double mutants could be observed protruding or blebbing into the central vacuole (Fig. 6c and d) similar to what has previously been observed in single *fc2-1* mutants [15]. Such structures were observed two times in 15 *fc2-1 atg5* cells, two times in 13 *fc2-1 atg7* cells, and zero times in 15 *fc2-1* cells. Together these results suggest *atg5* and *atg7* are unable reduce the number of degrading chloroplasts or to block chloroplast blebbing in the *fc2-1* mutant. In wt, *atg5*, and *atg7*, no chloroplast blebbing was observed and only a single degrading chloroplast was identified in *atg7*.

**Table 1:** Quantification of chloroplast degradation in four day old seedlings by transmission electron microscopy

genotype	cells imaged	chloroplasts imaged	degrading chloroplasts	% degrading chloroplasts
*wt*	7	31	0	0%
*atg5*	6	26	0	0%
*atg7*	7	29	1	3.4%
*fc2-1*	9	59	11	18.6%
*fc2-1 atg5*	9	59	23	39.0%
*fc2-1 atg7*	13	53	24	45.3%

In addition to increasing the percentage of degrading chloroplasts, the *atg* mutations also affected chloroplast ultrastructure in the *fc2-1* background. In many cases, *fc2-1 atg5* and *fc2-1 atg7* chloroplasts had irregular shapes or contained unusual horseshoe shaped membranes/vesicles (Fig. 6a and b). Such abnormalities were not observed in the single *atg5* and *atg7* mutants. The average chloroplast area was not reduced by the *atg* mutations in either the wt or *fc2-1* backgrounds, suggesting chloroplast development was not grossly delayed by the loss of core autophagy machinery (Fig. 6e). As such, ATG-dependent autophagy may still play a role in maintaining chloroplast structure and/or function during photo-oxidative stress.

The number of intact and degrading chloroplasts were quantified from transmission electron microscopy images taken of four-day-old seedlings. Degrading chloroplasts were identified by their abnormal shapes, severely disorganized internal membranes, and compressed thylakoids. Representative images of such chloroplasts are shown in Fig. 6b. Additional cells were monitored to identify blebbing chloroplasts, but were not included in this analysis.

### Genes encoding microautophagy-related proteins are induced in stressed *fc2* mutants

**Fig. 7:**
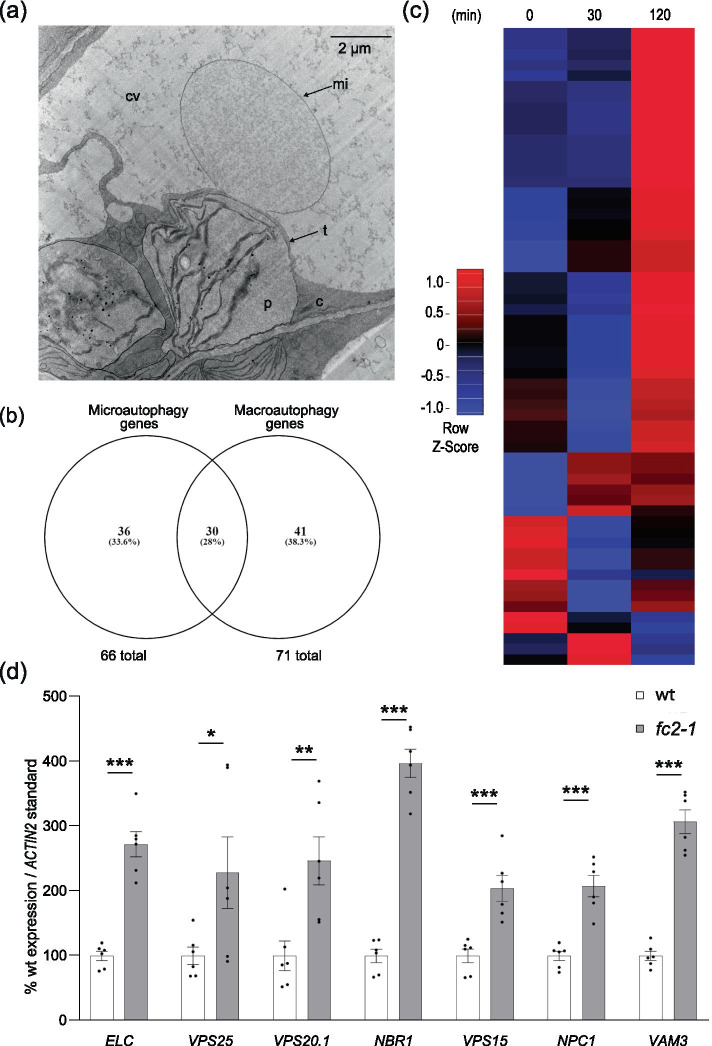
Microautophagy-related genes are transcriptionally induced in stressed *fc2* seedlings The induction of microautophagy was assessed in *fc2-1* seedlings. **A** Shown is a representative TEM image of the invagination of the vacuolar membrane associated next to a degrading chloroplast in the *fc2-1 atg5* mutant. c; cytoplasm, cv; central vacuole, mi; microautophagy structure, p; plastid, t; tonoplast. **B** A Venn diagram showing overlap of putative microautophagy-related genes and autophagy-related genes used in this study (Tables S1 and S2). **C** A heatmap of microautophagy-related gene expression (relative to wt) in etiolated seedlings at time points prior to (0 min) and during de-etiolation (30 min and 120 min). Microarray data was previously generated from etiolated seedlings grown in the dark for four days at time points before and after light exposure [15]. The list of genes considered is presented in Table S2. **D** RT-qPCR analysis of select microautophagy-related transcripts from four-day-old seedlings grown under 6 h light/18 h dark light cycling conditions. Shown are mean values ± SEM (n = 6 biological replicates). Statistical analyses were performed by student’s t-tests. *, **, *** indicate a p-value of ≤ 0.05, ≤ 0.01, and ≤ 0.001, respectively. In all bar graphs, closed circles represent individual data points

The observation that ^1^O_2_-mediated chloroplast degradation and blebbing into the central vacuole still occurs in *fc2-1 atg* double mutants suggests canonical autophagosome formation is dispensable for such a degradation pathway. This led us to consider alternative forms of autophagy that may play a role in ^1^O_2_-mediated selective chloroplast degradation. As the chloroplast blebbing observed in this process appears to involve invagination into the central vacuole (in the double mutants (Fig. 6c and d) and in *fc2-1* [15]), we aimed to investigate if ATG-independent microautophagy may play a role in this mechanism. Indeed such structures were also observed in *fc2-1 atg* double mutants that appeared independent of chloroplasts (Fig. 7a), suggesting microautophagy is still active in these lines. However, quantification of microautophagy or chloroplast blebbing events is difficult due to their infrequency in two-dimensional micrographs [15]. This is likely due to the small size of the connection point between the chloroplast bleb/vacuolar structure and the cytoplasm.

Therefore, to determine if microautophagy may be responsible for structures associated with chloroplast degradation in *fc2-1* mutants, we monitored the expression of genes that have been predicted to play a role in microautophagy due to their homology to microautophagy and microautophagy-related processes in *Saccharomyces cerevisiae* [34]. From this work, we generated a list of 66 genes to investigate in *fc2-1* (Table S2). As microautophagy itself is not well defined, particularly in plants [34], we first determined which genes this microautophagy gene list were shared with our manually curated list of general autophagy genes used in Fig. 1a and Table S1. There were 30 genes common to both lists, nearly all of which were the core ATG genes (Fig. 7b). As most of the predicted microautophagy-related genes were unique, we generated a heatmap using the same previously published microarray data set of etiolated wild type (wt) and *fc2-1* seedlings before and immediately after light exposure [15]. Of the 66 genes, 60 were included in the data set. At 120 min, 8 out of the 60 genes (13%) were significantly induced in *fc2-1* compared to wt (fold change ≥ 1.25, FDR ≤ 0.05). Again, a heatmap analysis showed a pattern of mild induction, suggesting this subset of predicted microautophagy-related genes are up-regulated in the *fc2-1* mutant, primarily 120 min after light exposure (Fig. 7c).

As these microarray data were from etiolated seedlings prior to and after light exposure, we again chose to validate these patterns in four day-old *fc2-1* seedlings grown under 6 h light/18 h dark light cycling conditions using RT-qPCR. Here, we probed selected genes from a number of functional gene categories [34]: ESCRT-I, *AT3G12400* (*ELC*); ESCRT-II, *AT4G19003* (*VPS25*); ESCRT-III, *AT5G63880* (*VPS20.1*); Vacuolar Selective Ubiquitin Receptor, *AT4G24690* (*NBR1*); PI3K Complex, *AT4G29380* (*VPS15*); Niemann–Pick type C proteins (NPC), *AT1G07230* (*NPC1*); SNARE, *AT5G46860* (*VAM3*)*.* In all cases, transcript abundance was significantly increased in *fc2-1* compared to wt. Together, these data suggest microautophagy is transcriptionally induced in *fc2-1* seedlings under ^1^O_2_-generating conditions. Based on the type of structures known to associate with ^1^O_2_-stressed chloroplasts, this may indicate a putative role for ATG-independent microautophagy in the mechanism by which ^1^O_2_-damaged chloroplasts are degraded in *fc2* mutants.

## Discussion

Chloroplast ROS accumulate under various environmental stresses and have the ability to initiate multiple signaling pathways. Usually, however, more than one type of ROS is generated simultaneously and it is difficult to understand the signaling contributions of each species [1, 56]. As such, the use of mutants that specifically and conditionally accumulate only one type of ROS have been very important for understanding the signaling abilities and mechanisms of individual species. The *fc2* mutant, which specifically accumulates chloroplast ^1^O_2_ under diurnal signaling conditions, has been an extremely useful system to dissect the role of this ROS in initiating chloroplast quality control and cellular degradation pathways. Genetic suppressor screens have identified chloroplast gene expression and the cellular ubiquitination machineries as playing important roles in these processes, providing insight into how ^1^O_2_ initiates a stress signal and how individual chloroplasts may be recognized for turnover [15, 16]. While such chloroplast degradation appears to be a targeted and orderly process, the cellular machinery involved has remained obscure. In order to further understand the mechanisms behind this degradation, here we tested the possibility that autophagy is involved in ^1^O_2_–mediated cell death pathways or in removing ^1^O_2_–damaged chloroplasts. Characterization of *fc2* suppressor (*fts*) mutations have suggested these two phenotypes are linked [15, 16].

Previous work has shown ATG-dependent autophagy can be involved in translocating UVB or EL-damaged chloroplasts to the central vacuole in a process referred to as chlorophagy [37, 39]. The autophagosome/chloroplast interaction differed under the two types of stresses. UVB caused a classic macroautophagy-like response with full envelopment of the damaged chloroplasts by the autophagosome [37]. EL, on the other hand, involved a microautophagy-like response with the autophagosome partly surrounding the damaged chloroplast, which was engulfed by the tonoplast [39]. In both cases, chlorophagy was entirely dependent on the core autophagy machinery and chloroplasts remained in the cytoplasm in *atg5* and *atg7* mutants. We hypothesized that if broad chlorophagy is responsible for cell death in *fc2* mutants grown under light cycling conditions, these *atg* mutations (similar to *fts* mutations) may suppress such cell death. However, when the same mutations (including a third mutation affecting core autophagy protein ATG10) were introduced into the *fc2* background, they did not suppress ^1^O_2_–induced cellular degradation. All three *fc2 atg* double mutants still experienced cell death as seedlings or adult plants when grown under diurnal cycling light (Figs. 3a and 4a). Taken together, these results demonstrate that chlorophagy is not directly involved in ^1^O_2_-induced cell death. Instead, the core autophagy machinery and macroautophagy were dispensable.

While ^1^O_2_-induced cellular degradation can occur in *fc2* mutants without core autophagy machinery, there still appears to be a relationship. Under diurnal cycling light conditions that produce chloroplast ^1^O_2_, *fc2* seedlings respond by transcriptionally inducing autophagy-related genes, including those encoding core autophagy proteins (Fig. 1a and d). Another induced autophagy-related gene, *BCL2-ASSOCIATED ATHANOGENE 6* (*BAG6*), is particularly interesting as it encodes a homolog of a mammalian regulator of apoptosis and is associated with the central vacuole. When overexpressed in Arabidopsis, it leads to necrotic lesions in leaves, suggesting a possible connection between selective chloroplast degradation and a vacuole-mediated cell death signal [41, 57, 58].

Autophagosomes containing ATG8a are also present in *fc2* cells, even though they do not appear to be directly interacting with chloroplasts for degradation (Fig. 2). Instead, such a phenotype may be related to an induced starvation response in *fc2* seedlings as evidenced by the induction of carbon and nitrogen starvation marker genes (Fig. 1e). It is possible the chloroplast dysfunction and cellular degradation in ^1^O_2_-stressed *fc2* seedlings have a pleotropic effect on cellular metabolism leading to the induction of autophagosomes and the remobilization of nutrients. As such, autophagosomes and the induction of general autophagy genes may be partly a consequence rather than the cause of the cellular degradation observed in *fc2* seedlings. Furthermore, ATG8 can also be part of an autophagy-independent proteolysis pathway, at least during senescence [59], suggesting other possibilities for these proteins.

It is tempting to conclude canonical autophagy is also dispensable for ^1^O_2_-induced chloroplast degradation. At least, such degradation occurs in the absence of autophagosomes. When *fc2 atg5* and *fc2 atg7* double mutants were grown under permissive 24 h constant light, they accumulated more degrading chloroplasts than *fc2*. Chloroplast protrusion/blebbing into the central vacuole, a characteristic of ^1^O_2_-mediated selective chloroplast degradation in *fc2* [15], was also still present in the double mutants. However, the *atg* mutations did affect how *fc2* chloroplasts responded to photo-oxidative stress, suggesting a complex relationship between autophagy and ^1^O_2_ stress. When grown for three days under light cycling conditions, the *atg5*, *atg7*, and *atg10* mutations appeared to slightly protect the *fc2* mutant from photoinhibition (increased F_v_/F_m_ values compared to *fc2-1*). However, compared to *fc2-1*, photosynthesis in the double mutants recovered more slowly over the following four days (Fig. 3e, and 3f). These effects on photosynthesis were dependent on the *fc2-1* background, as *atg* single mutants were almost indistinguishable from wt. Such a result suggests the loss of autophagy altered the sensitivities of chloroplasts to photo-oxidative damage. Indeed, an analysis of chloroplast ultrastructures demonstrated that chloroplasts in the double mutants were irregular in shape and contained unusual internal membrane structures (Fig. 6a and b). The number of degrading chloroplasts also appeared to increase in the double mutants compared to *fc2-1*. As such, autophagy may still be important for maintaining chloroplast homeostasis and function during ^1^O_2_ stress. This could be due for indirect reasons (e.g., nutrient remobilization or general cell health) or perhaps by directly regulating chloroplast proteins levels through autophagy-dependent protein quality control pathways. Such pathways can involve RuBisCO-containing bodies (RCB’s) [60] and ATG8-interacting protein (ATI) bodies [61] that package chloroplast proteins for degradation in the central vacuole in a piecemeal fashion. Therefore, a reduction of such autophagy may lead to an increase in degrading chloroplasts as observed in *fc2-1 atg* double mutants.

**Fig. 8:**
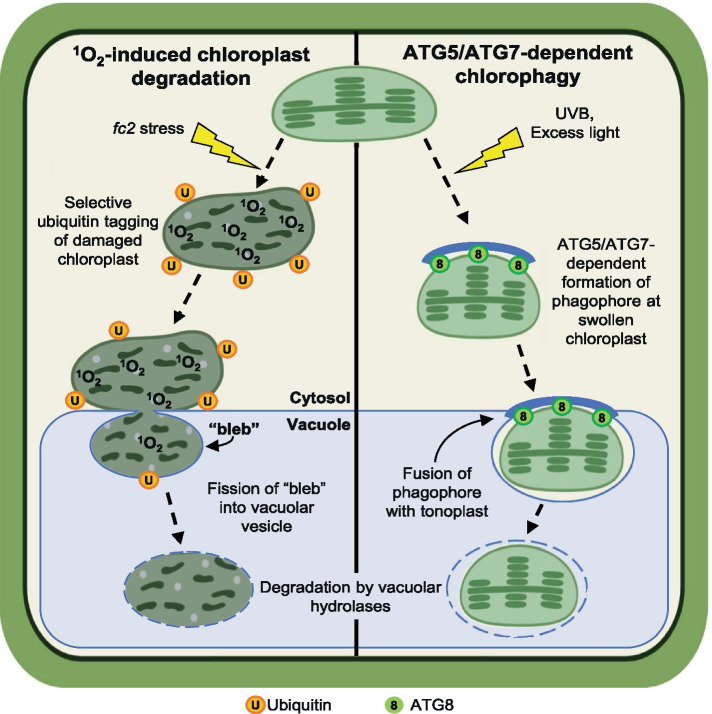
Model for two microautophagy-like processes for selective chloroplast turnover in plants. A hypothetical model depicting the proposed ^1^O_2_-induced chloroplast quality control [15] (left side) and ATG5/ATG7-dependent chlorophagy [38–40] (right side). In ^1^O_2_-induced chloroplast degradation, damaged chloroplasts are ubiquitin-tagged and begin degrading in the cytosol before being “blebbed” into the central vacuole for final turnover of chloroplast components. Vacuolar vesiculation of this bleb may proceed by “pinching off” via an autophagosome-independent fission-like mechanism [36]. Conversely, ATG5/ATG7-dependent chlorophagy, proceeds by a mechanism that requires phagophore formation and association with swollen, membrane damaged chloroplasts. Final vacuolar vesiculation in this process is likely achieved by a fusion of the chloroplast associated phagophore with the tonoplast membrane 36. In both processes, final degradation of chloroplast components are then degraded by vacuolar hydrolases

If chlorophagy and macroautophagy are not directly involved in ^1^O_2_–induced chloroplast degradation, then what process is being used? There are several differences between these processes indicating they represent fundamentally different types of events (Fig. 8). For instance, chlorophagy takes one or three days to be initiated after EL [38] and UVB stress [37], respectively. Conversely, ^1^O_2_–induced chloroplast degradation is initiated within three hours after photo-oxidative stress [15]. ^1^O_2_–induced chloroplast degradation in *fc2* cells is also visually distinct. Chloroplasts in *fc2* mutants already appear to be in a state of degradation before they protrude or “bleb” into the central vacuole without the obvious presence of autophagosome membranes (Fig. 6d and [15]). Such a structure is visually similar to microautophagy, involving the invagination of the central vacuole around cytosolic components [34, 62]. Unlike EL-induced chlorophagy, however, protrusion of ^1^O_2_–damaged chloroplasts into the central vacuole is independent of autophagosomes and the core autophagy machinery. As this was suggestive of ATG-independent microautophagy, we monitored the expression of predicted microautophagy-related genes and found a clear pattern of induction in the *fc2* mutant (Fig. 7d).

Although several specific types of microautophagy exist, it has been proposed that it can proceed by two general methods in yeast and animal cells: fusion-type and fission-type microautophagy [36]. Fusion-type microautophagy depends on core ATGs and partial autophagosome formation that eventually fuses with the lysosome membrane upon final vesiculation [36]. Fission-type microautophagy on the other hand, is independent of the core autophagy machinery. Instead, it involves a recruitment of endosomal sorting complex required for transport (ESCRT) proteins that lease to a “pinching off” of lysosomal invaginations to form an intra-lysosomal vesicle [36]. These two methods of vacuolar transport, if conserved in plants and applicable to the central vacuole, correlate well with the two different mechanisms of vacuolar transport observed during chlorophagy [39] (fusion-type) and ^1^O_2_-induced chloroplast degradation ([15] and this study) (fission type). As such, we currently favor the hypothesis that such an ATG-independent microautophagy may be specifically involved in removing ^1^O_2_–damaged chloroplasts from the cell. Currently, however, there are no clear ways to specifically and unambiguously block these microautophagy pathways, making their study quite challenging [36].

In yeast, such fission-type ATG-independent microautophagy is involved in the degradation of endoplasmic reticulum components (Micro-ER-phagy) [63] and lipid droplets [64, 65]. In Arabidopsis, this type of microautophagy may be involved in the transport of anthocyanin pigment clusters from the cytoplasm to the vacuole [66]. In this case, the tonoplast protrudes into the cytoplasm to make contact with the cargo, which may make it distinct from the process behind the degradation of ^1^O_2_-damaged chloroplasts. Further examination of the mechanisms behind selective vacuolar transport of ^1^O_2_-damaged chloroplasts in *fc2* mutants may help to define the pathways involved in this poorly understood form of autophagy.

## Conclusions

In this work, we have demonstrated that ^1^O_2_-induced cellular degradation and selective chloroplast degradation in the *fc2* mutant occur via a process distinct from chlorophagy and can proceed in the absence of ATG-dependent autophagy or the core autophagy machinery (Fig. 8). This is supported by a previous report demonstrating the PUB4 protein, which is required for selective chloroplast degradation in the *fc2* mutant [15], is dispensable for chlorophagy after EL stress [40]. Together, these results suggest alternate pathways of chloroplast degradation can be used in the cell, possibly depending on the type of stress or damage being experienced. Such a model would explain the altered starvation responses and severe early senescence phenotypes of *pub4 atg5* and *pub4 atg7* double mutants that may lack two independent pathways for dismantling chloroplasts [40]. Further work will still be necessary to understand the different roles of these pathways during stress, how they recognize damaged chloroplasts, and how these chloroplasts are ultimately transported to the central vacuole. A deeper understanding of the mechanisms behind these pathways will be of central importance in understanding how cells maintain chloroplast function and homeostasis under dynamic and stressful conditions and may help to provide molecular tools to control photosynthesis and yield in agriculturally important crop species.

## Methods

### Biological material, growth conditions, and treatments

No permissions were necessary to collect plant material. *Arabidopsis thaliana* ecotype *Columbia* (Col-0) stored in our laboratory was the wild type (wt) line used in this study. T-DNA lines GABI_766H08 (*fc2-1*) and GABI_655B06 (*atg7-2*) from the GABI-Kat collection [67], SALK_084434 (*atg10-1*) from the SALK (The Salk Institute Genomic Analysis Laboratory) collection [68] and SAIL_129_B07 (*atg5-1*) from the SAIL collection [69] were all obtained from the Arabidopsis Biological Resource Center (Columbus, OH) and described previously [33, 50, 52, 70]. The mutants *pub4-6* and *toc33* are stored in our laboratory and were described previously [15]. Additional information is listed in Table S3. Double mutant lines were generated via crossing and confirmed by extracting genomic DNA (following a CTAB-based protocol [71]) and confirming genotypes using PCR-based markers (Fig. S2a) (primers listed in Table S4).

Seeds used in growth experiments were sterilized overnight using chloride gas and then resuspended in a 0.1% agar solution. Resuspended seeds were then spread on plates containing Linsmaier and Skoog medium pH 5.7 (Caisson Laboratories North Logan, UT) in 0.6% micropropagation type-1 agar powder. Plates were then stratified for three-five days and then germinated and grown in constant light (24 h day) or diurnal cycling light (6 h day/18 h night) conditions at a light intensity of ~ 100 photons µmol m^−2^ s^−1^ at a temperature of 21 °C. For adult plant experiments, seven-day old seedlings grown in constant light conditions were carefully transferred to soil and growth was continued under similar conditions in a reach-in plant growth chamber. For dark starvation experiments, plates containing four day old seedlings were wrapped in aluminum foil three times and then placed back into the growth chamber. Six days later, these plates were opened under dim light and the seedlings were imaged by confocal microscopy. Photosynthetically active radiation was measured using a LI-250A light meter with a LI-190R-BNC-2 Quantum Sensor (LiCOR).

*Agrobacterium tumefaciens* was grown in liquid Miller nutrient broth or solid medium containing 1.5% agar (w/v). Cells were grown at 28 °C with the appropriate antibiotics and liquid medium was shaken at 225 rpm.

### Construction of transgenic Arabidopsis lines

The vector containing the *UBQ10*::*GFP-ATG8a* construct (At4g21980.1 [72]) was transformed into the *A. tumefaciens* strain GV3101, which was subsequently used to transform Arabidopsis via the floral dip method. T_1_ plants were selected for their ability to grow on Basta-soaked soil and propagated to the next generation. T_2_ lines were monitored for single insertions (segregating 3:1 for Basta resistance: sensitivity) and propagated to the next generation. Finally, homozygous lines were selected in the T_3_ generation based on 100% Basta-resistance.

### Confirmation of T-DNA mutant lines by PCR genotyping

Primers were designed using the SIGnAL (http://signal.salk.edu/) T-DNA primer design tool. Primers for *fc2-1*, *atg5-1*, *atg7-2*, and *atg10-1*, were used to confirm the genotypes of single and double mutants (Table S4 and Fig. S2a). All PCR was performed using Promega GoTaq® Master Mix, with an initial denaturation step at 93 °C for 3 min, followed by a 30 s denaturation step at 93 °C, a 30 s annealing step at 60 °C, and a 1 min 15 s elongation step at 72 °C, cycled 35 times and then concluded with a final elongation step at 72 °C for 7 min. These thermocycler settings were used for all primer sets, excepting LB + RP:JP286/1169 for *atg7-2,* which required a lower annealing temp (54 °C) to achieve amplification. This resulted in DNA fragments between ~ 0.5–1.0 kb in length, which were separated on a 1% agarose gel for imaging.

### Biomass measurements

For dry weight (DW) biomass measurements, eight replicates of each line and growth condition were weighed then stored in envelopes for desiccation. Tissue was desiccated in a 65 °C oven for 48 h and then remeasured to record dry weight.

### Chlorophyll fluorescence measurements

To measure Maximum PSII quantum yield (F_v_/F_m_), seedlings were germinated under 24 h constant or 6 h/18 h light/dark cycling light conditions. F_v_/F_m_ was measured two hours after subjective dawn every day for six days. Plates were placed in the FluorCam chamber (Closed FluorCam FC 800-C/1010-S, Photon Systems Instruments) for 15 min to dark acclimate without a plate cover, and then the measurements were taken following the manual provided by the manufacturer. Each genotype was sown in triplicate, on separate plates, and F_v_/F_m_ measurements were taken from each replicate consecutively.

### Chlorophyll measurements

Total chlorophyll was extracted using 100% ethanol from seven-day-old seedlings. Cell debris was pelleted thrice at 12,000 × g for 30 min at 4 °C. Chlorophyll was measured spectrophotometrically in 100 μl volumes in a Biotek Synergy H1 Hybrid Reader and path corrections were calculated according to [73]. Chlorophyll levels were normalized per seedling (~ 50–130 per line and counted prior to germination).

### Cell death measurements

Cell death was assessed using trypan blue staining as previously described [16]. Briefly, seedings were collected at seven days and then transferred to a solution of Trypan Blue (glycerol: 1.25 ml, trypan blue solution: 0.625 ml, ddH_2_O: 0.625 ml, lactic acid: 1.25 ml, phenol: 1.25 ml, 100% ethanol: 10 ml). The solution was then boiled for 1 min, incubated overnight, and then destined with a solution of saturated chloral hydrate (2.5 g per 1 ml water) Chloral hydrate was then removed, and seedlings were stored in 30% (v:v) glycerol solution prior to imaging on a dissection scope. For each replicate, staining intensity was measured across one entire cotyledon (seedlings) or leaf (adult plants) using ImageJ software.

### RNA extraction and RT-qPCR

Total RNA was extracted from whole seedlings using the RNeasy Plant Mini Kit (Qiagen) and cDNA was synthesized using the Maxima first-strand cDNA synthesis kit for RT-qPCR with DNase (Thermo Scientific) following the manufacturer's instructions. Real-time PCR was performed using the iTAQ Universal SYBR Green Supermix (BioRad) with the SYBR Green fluorophore and a CFX Connect Real-Time PCR Detection System (Biorad). The following 2-step thermal profile was used in all RT-qPCR: 95 °C for 3 min, 40 cycles of 95 °C for 10 s, and 60 °C for 30 s (as per the manufacturer’s instructions). All expression data was normalized according to *ACTIN2* expression. All primers used for RT-qPCR are listed in Table S4.

### Transmission electron microscopy

Four day old seedlings were prepared and imaged by transmission electron microscopy as previously described [16]. Chloroplast area was analyzed using ImageJ.

### In vivo protein localization

Four day-old seedlings (or 10 day old seedlings for dark starvation experiments) were imaged on a Zeiss 880 inverted confocal microscope. Seedlings were carefully placed on glass slides with half-strength liquid LS media and carefully overlaid with a coverslip (no. 1.5). Chlorophyll autofluorescence and GFP were monitored using separate tracks to maximize fluorescence detection and eliminate potential crosstalk. GFP was excited with a 488-nm laser and emission data was collected between 500–570 nm. Chlorophyll was excited with a 633-nm laser and emission data was collected between 640–720 nm. To test for fluorescence bleed-through, untransformed *fc2-1* seedlings from each condition were imaged under identical settings and shown in Fig. 2. Minimal background green fluorescence was detected using the 488-nm laser. Seedlings were imaged using the 63 × objective, with an additional 2 × or 3 × digital magnification. Resulting images were processed using Zen Blue (Carl Zeiss) software. As different light treatments affected chlorophyll levels, chlorophyll autofluorescence was adjusted in each sample to clearly identify the chloroplast organelles. Experiments were performed three times and representative images are shown. Plot profile analyses were performed with ImageJ/Fiji.

### Gene expression meta-analysis

To monitor the expression of autophagy-related genes controlled by chloroplast ^1^O_2_ signals, we used a previously published data set of a microarray analysis of four-day-old etiolated wt and *fc2-1* seedlings before and after (30 and 120 min) light exposure (120 µmol photons m^−2^ s^−1^) [15]. Differential gene expression and FDR values were produced using the edgeR differential gene expression package on https://galaxyproject.org/ [74, 75]. Manually curated list core autophagy and autophagy related genes (Table S1) were compiled from gene ontology biological process term GO:0016238 from the Arabidopsis Information Resource (TAIR) website (https://www.arabidopsis.org/). Parent (24 loci) and children terms (60 loci) were collected. Duplicate and undefined loci were removed. A list of putative microautophagy-related genes (Table S2) and associated loci were compiled from [34], and additional information was obtained from TAIR. A data matrix reflecting the microarray expression data was then compiled for each of these lists. Genes with no detected expression data or no associated microarray probe were removed from the data matrix. Heatmaps were generated using the heatmapper package (https://github.com/WishartLab/heatmapper) on http://www.heatmapper.ca/ [76]. Average linkage was used for the clustering method and Pearson correlation was used for the distance measurement method. Venn diagrams were generated using Venny Diagram (https://bioinfogp.cnb.csic.es/tools/venny/) [77].

### Graphical model creations

The model depicting chloroplast degradation pathways in Fig. 8 was created using online BioRender software (https://biorender.com/).

## Supplementary Information

**Additional file 1: FigureS1.** Co-localization of GFP-ATG8a and chloroplasts. **FigureS2.** Validation of *atg5*, *atg7*, and *atg10* null mutationsin the *fc2-1* background. **Figure S3.** Phenotypes of* atg5*, *atg7*,and *atg10* single mutant seedlings. **Figure S4.** Assessment of photosynthetic efficiency in the *atg*single and *fc2 atg* double mutants. **Figure S5.** Phenotypes of* atg5*, *atg7*, and *atg10*single mutant adult plants.

**Additional file 2: Table S1.**List of autophagy-related genes and expression data used in heatmap generation.**Table S2.** List of predicted microautophagy-related genes and expressiondata used in heatmap generation. **Table S3.** Mutants used in this study. **TableS4.** Primers use in this study.

## Data Availability

The microarray data set analyzed in this study has been submitted to the Gene Expression Omnibus database with accession no. GSE71764 (https://www.ncbi.nlm.nih.gov/geo/query/acc.cgi?acc=GSE71764). All other data generated and analyzed during this study are included in this published article and its supplementary information files.

## Abbreviations

^1^O_2_: Singlet oxygen
QC: Quality control
ROS: Reactive oxygen species

## References

[1] Asada K (2006). Production and scavenging of reactive oxygen species in chloroplasts and their functions. Plant Physiol.

[2] Poljsak B, Šuput D, Milisav I. Achieving the balance between ROS and antioxidants: When to use the synthetic antioxidants. Oxid Med Cell Longev. 2013;2013:956792. 10.1155/2013/956792 PMC3657405 23738047

[3] Müller P, Li XP, Niyogi KK. Non-photochemical quenching. A response to excess light energy. Plant Physiol. 2001;125(4):1558–66. 10.1104/pp.125.4.1558 PMC1539381 11299337

[4] Horton P, Ruban A (2005). Molecular design of the photosystem II light-harvesting antenna: Photosynthesis and photoprotection. J Exp Botany.

[5] Triantaphylidès C, Krischke M, Hoeberichts FA, Ksas B, Gresser G, Havaux M (2008). Singlet oxygen is the major reactive oxygen species involved in photooxidative damage to plants. Plant Physiol.

[6] Chan KX, Mabbitt PD, Phua SY, Mueller JW, Nisar N, Gigolashvili T (2016). Sensing and signaling of oxidative stress in chloroplasts by inactivation of the SAL1 phosphoadenosine phosphatase. Proc Natl Acad Sci U S A.

[7] Suo J, Zhao Q, David L, Chen S, Dai S (2017). Salinity response in chloroplasts: Insights from gene characterization. Int J Mol Sci.

[8] Lu Y, Yao J (2018). Chloroplasts at the crossroad of photosynthesis, pathogen infection and plant defense. Int J Mol Sci.

[9] De Souza A, Wang JZ, Dehesh K (2017). Retrograde Signals: Integrators of Interorganellar Communication and Orchestrators of Plant Development. Ann Rev Plant Biol.

[10] Op Den Camp RGL, Przybyla D, Ochsenbein C, Laloi C, Kim C, Danon A, et al. Rapid Induction of Distinct Stress Responses after the Release of Singlet Oxygen in *Arabidopsis*. Plant Cell. 2003;15(10):2320–32. 10.1105/tpc.014662 PMC197298 14508004

[11] Dogra V, Kim C (2020). Singlet Oxygen Metabolism: From Genesis to Signaling. Front Plant Sci.

[12] Woodson JD (2019). Chloroplast stress signals: regulation of cellular degradation and chloroplast turnover. Curr Opin Plant Biol.

[13] Scharfenberg M, Mittermayr L, Von Roepenack-Lahaye E, Schlicke H, Grimm B, Leister D (2015). Functional characterization of the two ferrochelatases in *Arabidopsis thaliana*. Plant Cell Environ.

[14] Fernandez JM, Bilgin MD, Grossweiner LI (1997). Singlet oxygen generation by photodynamic agents. J Photochem Photobiol B Biol.

[15] Woodson JD, Joens MS, Sinson AB, Gilkerson J, Salomé PA, Weigel D (2015). Ubiquitin facilitates a quality-control pathway that removes damaged chloroplasts. Science.

[16] Alamdari K, Fisher KE, Sinson AB, Chory J, Woodson JD (2020). Roles for the chloroplast-localized pentatricopeptide repeat protein 30 and the ‘mitochondrial’ transcription termination factor 9 in chloroplast quality control. Plant J.

[17] González-Pérez S, Gutiérrez J, García-García F, Osuna D, Dopazo J, Lorenzo Ó (2011). Early transcriptional defense responses in arabidopsis cell suspension culture under high-light conditions. Plant Physiol.

[18] Ogilby PR (2010). Singlet oxygen: There is indeed something new under the sun. Chem Soc Rev.

[19] Dmitrieva VA, Tyutereva EV, Voitsekhovskaja OV (2020). Singlet oxygen in plants: Generation, detection, and signaling roles. Int J Mol Sci.

[20] Wagner D, Przybyla D, Op Den Camp R, Kim C, Landgraf F, Keun PL, et al. The genetic basis of singlet oxygen-induced stress response of *Arabidopsis thaliana*. Science. 2004;306(5699):1183–5. 10.1126/science.1103178 15539603

[21] Barkan A, Small I (2014). Pentatricopeptide repeat proteins in plants. Ann Rev Plant Biol.

[22] Méteignier LV, Ghandour R, Zimmerman A, Kuhn L, Meurer J, Zoschke R (2021). *Arabidopsis* mTERF9 protein promotes chloroplast ribosomal assembly and translation by establishing ribonucleoprotein interactions in vivo. Nucleic Acids Res.

[23] Stephani M, Dagdas Y (2020). Plant Selective Autophagy—Still an Uncharted Territory With a Lot of Hidden Gems. J Mol Biol.

[24] Izumi M, Nakamura S, Li N (2019). Autophagic turnover of chloroplasts: Its roles and regulatory mechanisms in response to sugar starvation. Front Plant Sci.

[25] King JS. Autophagy across the eukaryotes: Is *S. cerevisiae* the odd one out? Autophagy. 2012;8(7):1159–62. 10.4161/auto.20527 PMC3429559 22722653

[26] Anding AL, Baehrecke EH (2017). Cleaning House: Selective Autophagy of Organelles. Dev Cell.

[27] Pickles S, Vigié P, Youle RJ (2018). Mitophagy and Quality Control Mechanisms in Mitochondrial Maintenance. Curr Biol.

[28] Bassham DC, Laporte M, Marty F, Moriyasu Y, Ohsumi Y, Olsen LJ (2006). Autophagy in development and stress responses of plants. Autophagy.

[29] Liu Y, Bassham DC (2012). Autophagy: Pathways for self-eating in plant cells. Ann Rev Plant Biol.

[30] Wen X, Klionsky DJ. An overview of macroautophagy in yeast. J Mol Biol. 2016;428(9 pt A):1681–99. 10.1016/j.jmb.2016.02.021 PMC4846508 26908221

[31] Chun Y, Kim J (2018). Autophagy: An Essential Degradation Program for Cellular Homeostasis and Life. Cells.

[32] Yang Z, Klionsky DJ (2009). An overview of the molecular mechanism of autophagy. Curr Topics Microbiol Immunol.

[33] Hofius D, Munch D, Bressendorff S, Mundy J, Petersen M (2011). Role of autophagy in disease resistance and hypersensitive response-associated cell death. Cell Death Differentiation.

[34] Sieńko K, Poormassalehgoo A, Yamada K, Goto-Yamada S (2020). Microautophagy in Plants: Consideration of Its Molecular Mechanism. Cells.

[35] Su T, Li X, Yang M, Shao Q, Zhao Y, Ma C (2020). Autophagy: An Intracellular Degradation Pathway Regulating Plant Survival and Stress Response. Front Plant Sci.

[36] Schuck S. Microautophagy – distinct molecular mechanisms handle cargoes of many sizes. J Cell Sci. 2020;133(17):jcs246322. 10.1242/jcs.246322 32907930

[37] Izumi M, Ishida H, Nakamura S, Hidema J (2017). Entire photodamaged chloroplasts are transported to the central vacuole by autophagy. Plant Cell.

[38] Izumi M, Nakamura S (2017). Vacuolar digestion of entire damaged chloroplasts in *Arabidopsis thaliana* is accomplished by chlorophagy. Autophagy.

[39] Nakamura S, Hidema J, Sakamoto W, Ishida H, Izumi M (2018). Selective elimination of membrane-damaged chloroplasts via microautophagy. Plant Physiol.

[40] Kikuchi Y, Nakamura S, Woodson JD, Ishida H, Ling Q, Hidema J (2020). Chloroplast autophagy and ubiquitination combine to manage oxidative damage and starvation responses. Plant Physiol.

[41] Kang CH, Jung WY, Kang YH, Kim JY, Kim DG, Jeong JC (2006). AtBAG6, a novel calmodulin-binding protein, induces programmed cell death in yeast and plants. Cell Death Differentiation.

[42] Ren C, Liu J, Gong Q (2014). Functions of autophagy in plant carbon and nitrogen metabolism. Front Plant Sci.

[43] Fujiki Y, Yoshikawa Y, Sato T, Inada N, Ito M, Nishida I (2001). Dark-inducible genes from *Arabidopsis thaliana* are associated with leaf senescence and repressed by sugars. Physiol Plant.

[44] Gonzali S, Loreti E, Solfanelli C, Novi G, Alpi A, Perata P (2006). Identification of sugar-modulated genes and evidence for in vivo sugar sensing in *Arabidopsis*. J Plant Res.

[45] Lezhneva L, Kiba T, Feria-Bourrellier AB, Lafouge F, Boutet-Mercey S, Zoufan P, et al. The *Arabidopsis* nitrate transporter NRT2.5 plays a role in nitrate acquisition and remobilization in nitrogen-starved plants. Plant J. 2014;80(2):230–41. 10.1111/tpj.12626 25065551

[46] Yang M, Bu F, Huang W, Chen L (2019). Multiple regulatory levels shape autophagy activity in plants. Front Plant Sci.

[47] Yoshimoto K, Hanaoka H, Sato S, Kato T, Tabata S, Noda T (2004). Processing of ATG8s, ubiquitin-like proteins, and their deconjugation by ATG4s are essential for plant autophagy. Plant Cell.

[48] Wada S, Ishida H, Izumi M, Yoshimoto K, Ohsumi Y, Mae T (2009). Autophagy plays a role in chloroplast degradation during senescence in individually darkened leaves. Plant Physiol.

[49] Ishida H, Yoshimoto K, Izumi M, Reisen D, Yano Y, Makino A (2008). Mobilization of Rubisco and stroma-localized fluorescent proteins of chloroplasts to the vacuole by an ATG gene-dependent autophagic process. Plant Physiol.

[50] Thompson AR, Doelling JH, Suttangkakul A, Vierstra RD (2005). Autophagic nutrient recycling in *Arabidopsis* directed by the ATG8 and ATG12 conjugation pathways. Plant Physiol.

[51] Doelling JH, Walker JM, Friedman EM, Thompson AR, Vierstra RD (2002). The APG8/12-activating enzyme APG7 is required for proper nutrient recycling and senescence in *Arabidopsis thaliana*. J Biol Chem.

[52] Phillips AR, Suttangkakul A, Vierstra RD (2008). The ATG12-conjugating enzyme ATG10 is essential for autophagic vesicle formation in *Arabidopsis thaliana*. Genetics.

[53] Hofius D, Schultz-Larsen T, Joensen J, Tsitsigiannis DI, Petersen NHT, Mattsson O (2009). Autophagic Components Contribute to Hypersensitive Cell Death in *Arabidopsis*. Cell.

[54] Maxwell K, Johnson GN (2000). Chlorophyll fluorescence - A practical guide. J Exp Botany.

[55] Baruah A, Šimková K, Hincha DK, Apel K, Laloi C (2009). Modulation of 1O2-mediated retrograde signaling by the PLEIOTROPIC RESPONSE LOCUS 1 (PRL1) protein, a central integrator of stress and energy signaling. Plant J.

[56] Rosenwasser S, Fluhr R, Joshi JR, Leviatan N, Sela N, Hetzroni A (2013). ROSMETER: A bioinformatic tool for the identification of transcriptomic imprints related to reactive oxygen species type and origin provides new insights into stress responses. Plant Physiol.

[57] Li Y, Kabbage M, Liu W, Dickman MB (2016). Aspartyl protease-mediated cleavage of BAG6 is necessary for autophagy and fungal resistance in plants. Plant Cell.

[58] Kabbage M, Kessens R, Dickman MB (2016). A plant Bcl-2-associated athanogene is proteolytically activated to confer fungal resistance. Microbial Cell.

[59] Jia M, Liu X, Xue H, Wu Y, Shi L, Wang R (2019). Noncanonical ATG8–ABS3 interaction controls senescence in plants. Nat Plants.

[60] Chiba A, Ishida H, Nishizawa NK, Makino A, Mae T (2003). Exclusion of Ribulose-1,5-bisphosphate Carboxylase/oxygenase from Chloroplasts by Specific Bodies in Naturally Senescing Leaves of Wheat. Plant Cell Physiol.

[61] Michaeli S, Honig A, Levanony H, Peled-Zehavi H, Galili G (2014). *Arabidopsis* ATG8-INTERACTING PROTEIN1 is involved in autophagy-dependent vesicular trafficking of plastid proteins to the vacuole. Plant Cell.

[62] Van Doorn WG, Papini A (2013). Ultrastructure of autophagy in plant cells: A review. Autophagy.

[63] Schuck S, Gallagher CM, Walter P (2014). ER-phagy mediates selective degradation of endoplasmic reticulum independently of the core autophagy machinery. J Cell Sci.

[64] Oku M, Maeda Y, Kagohashi Y, Kondo T, Yamada M, Fujimoto T (2017). Evidence for ESC RT- and clathrin-dependent microautophagy. J Cell Biol.

[65] Iwama R, Ohsumi Y. Analysis of autophagy activated during changes in carbon source availability in yeast cells. J Biol Chem. 2019;294(14):5590–603. 10.1074/jbc.RA118.005698 PMC6462502 30755486

[66] Chanoca A, Kovinich N, Burkel B, Stecha S, Bohorquez-Restrepo A, Ueda T (2015). Anthocyanin vacuolar inclusions form by a microautophagy mechanism. Plant Cell.

[67] Kleinboelting N, Huep G, Kloetgen A, Viehoever P, Weisshaar B. GABI-Kat SimpleSearch: New features of the *Arabidopsis thaliana* T-DNA mutant database. Nucleic Acids Res. 2012;40(Database issue):D1211–5. 10.1093/nar/gkr1047 PMC3245140 22080561

[68] Alonso JM, Stepanova AN, Leisse TJ, Kim CJ, Chen H, Shinn P (2003). Genome-wide insertional mutagenesis of *Arabidopsis thaliana*. Science.

[69] Sessions A, Burke E, Presting G, Aux G, McElver J, Patton D (2002). A high-throughput *Arabidopsis* reverse genetics system. Plant Cell.

[70] Woodson JD, Perez-Ruiz JM, Chory J (2011). Heme synthesis by plastid ferrochelatase I regulates nuclear gene expression in plants. Current Biol.

[71] Healey A, Furtado A, Cooper T, Henry RJ (2014). Protocol: A simple method for extracting next-generation sequencing quality genomic DNA from recalcitrant plant species. Plant Methods.

[72] Nakamura S, Hagihara S, Otomo K, Ishida H, Hidema J, Nemoto T (2021). Autophagy Contributes to the Quality Control of Leaf Mitochondria. Plant and Cell Physiol.

[73] Warren CR (2008). Rapid measurement of chlorophylls with a microplate reader. J Plant Nutr.

[74] Robinson MD, McCarthy DJ, Smyth GK (2009). edgeR: A Bioconductor package for differential expression analysis of digital gene expression data. Bioinformatics.

[75] Afgan E, Baker D, Batut B, Van Den Beek M, Bouvier D, Ech M (2018). The Galaxy platform for accessible, reproducible and collaborative biomedical analyses: 2018 update. Nucleic Acids Res.

[76] Babicki S, Arndt D, Marcu A, Liang Y, Grant JR, Maciejewski A (2016). Heatmapper: web-enabled heat mapping for all. Nucleic Acids Res.

[77] Oliveros JC. VENNY. An interactive tool for comparing lists with Venn Diagrams. http://bioinfogp.cnb.csic.es/tools/venny/index.html. Accessed 14 July 2021.

