# Supplementary material for: The core autophagy machinery is not required for chloroplast singlet oxygen-mediated cell death in the Arabidopsis thaliana plastid ferrochelatase two mutant
Source: BMC Plant Biol. 2021 Jul 19;21:342. doi: 10.1186/s12870-021-03119-x (PMC8290626; doi:10.1186/s12870-021-03119-x)
Supplement: Supplementary file 1 — Additional file 1: FigureS1. Co-localization of GFP-ATG8a and chloroplasts. FigureS2. Validation of atg5, atg7, and atg10 null mutationsin the fc2-1 background. Figure S3. Phenotypes of atg5, atg7,and atg10 single mutant seedlings. Figure S4. Assessment of photosynthetic efficiency in the atgsingle and fc2 atg double mutants. Figure S5. Phenotypes of atg5, atg7, and atg10single mutant adult plants. [file 12870_2021_3119_MOESM1_ESM.pdf]

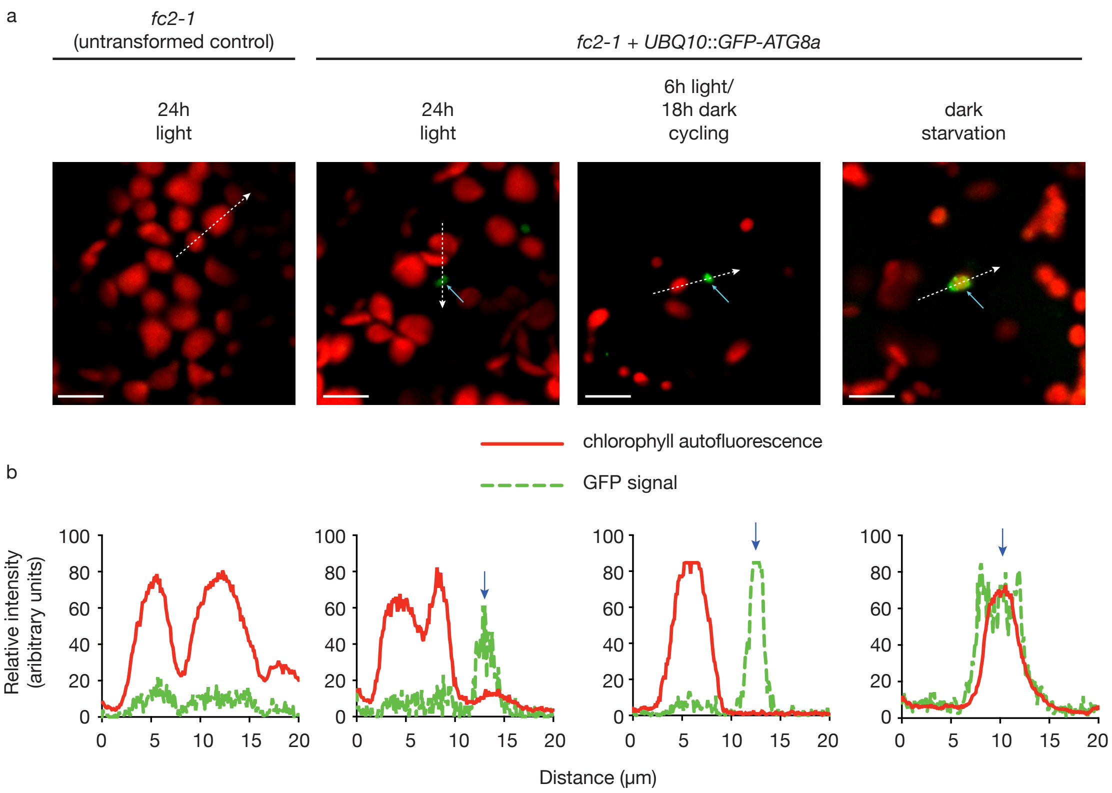

<u>Figure S1. Co-localization of GFP-ATG8a and chloroplasts.</u>
The co-localization of GFP-ATG8a and chlorophyll was assessed in the images shown in Fig. 2. **A)** Confocal images of chlorophyll (red) and GFP (green) overlay from Fig. 2. The genotype and growth conditions are indicated. Dashed white arrows indicate the 15 µm lines where fluorescence intensity was measured. Solid blue arrows indicate site of GFP accumulation. Solid white bars = 10 µm. **B)** Plotted fluorescence intensity (arbitrary units) of above images. Blue arrows indicate sire of observed GFP accumulation.

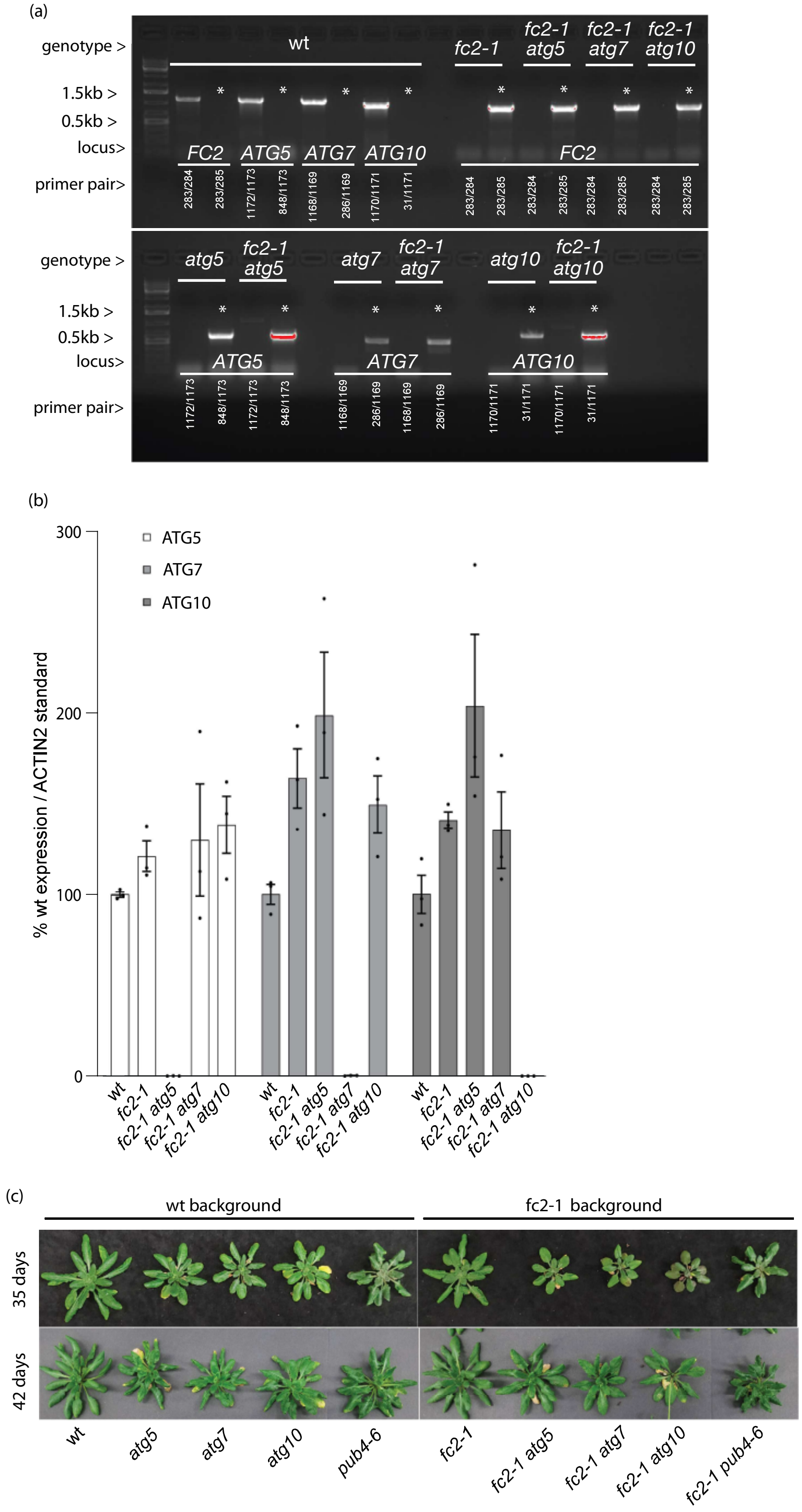

Figure S2. Validation of *atg5*, *atg7*, and *atg10* null mutations in the *fc2-1* background.

Molecular and physiological phenotypes of the *fc2-1 atg* mutants were assessed. **A)** Gel of PCR-based genotyping of single and double T-DNA mutants used in study. * indicates PCR testing for presence of T-DNA insertion. Primers used: wt *FC2*; JP283/284, *fc2-1*; JP283/285; wt *ATG5*; JP1172/1173, *atg5-1*; JP848/1173, wt *ATG7*; JP1168/1169; *atg7-2*; JP286/1169, wt *ATG10*; JP1170/1171, *atg10-1*; JP31/1171. Image is cropped from one large single DNA gel. **B)** RT-qPCR analysis of transcripts from four-day-old seedlings grown under 6h light/18h dark light cycling conditions. Shown are mean values +/- SEM (n = 3 biological replicates). Closed circles represent individual data points. Statistical tests were not performed. **C)** Images of representative 35 and 42 day old plants grown in 24h constant light conditions.

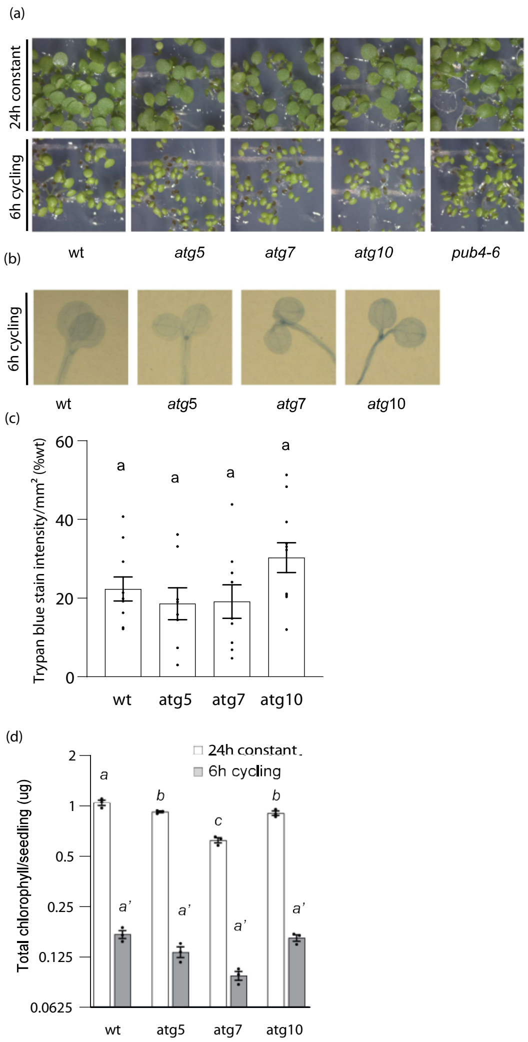

Figure S3. Phenotypes of *atg5*, *atg7*, and *atg10* single mutant seedlings.
The phenotypes of *atg* single mutant seedling were assessed in (24h) constant light and 6h light/18h dark (6h) cycling light. **A)** Seven-day old seedlings grown in 24h or 6h light conditions. **B)** Representative trypan blue stains of seedlings from panel A. The lack of dark blue color is indicative of healthy and alive cells. **C)** Mean values (+/- SEM) of the trypan blue signal in panel B ($n \geq 10$ seedlings). **D)** Mean total chlorophyll content (+/- SEM) of six-day old seedlings grown in 24h light or 6h cycling light conditions ($n = 3$ biological replicates). Statistical analyses were performed by one-way AVOVA tests followed by Tukey's HSD. Different letters above bars indicate significant differences (p value ≤ 0.05). For panel D, separate statistical analyses were performed for the different light treatments and the significance for the 6h cycling values is denoted by letters with a ′. In all bar graphs, closed circles represent individual data points.

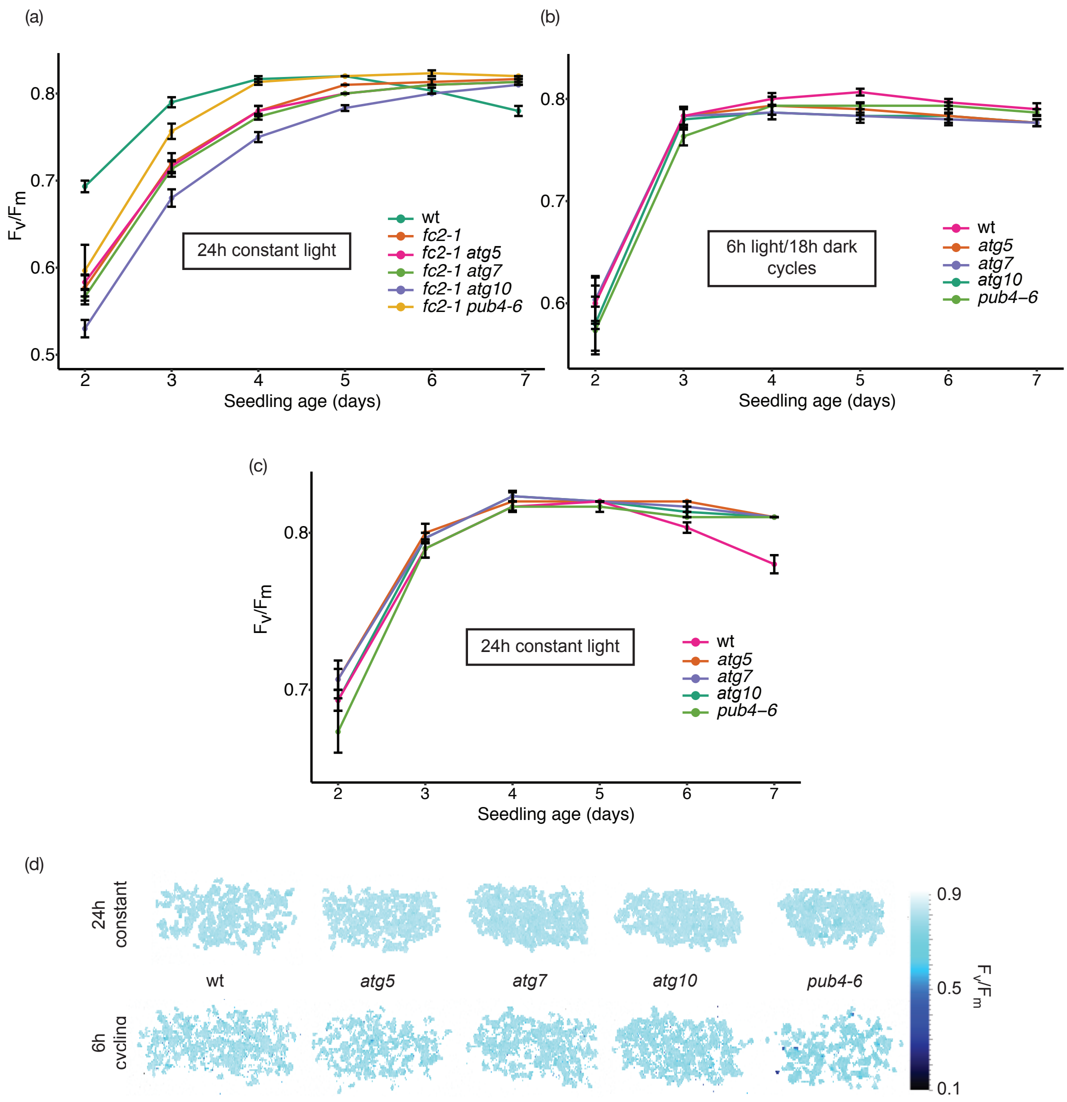

<u>Figure S4. Assessment of photosynthetic efficiency in the *atg* single and *fc2 atg* double mutants.</u> Maximum quantum yield of PSII (F_v/F_m) was measured in two to seven day old seedlings. **A)** Shown are the F_v/F_m values of seedlings in the *fc2-1* background grown under 24h constant light conditions and **B)** seedlings in the wt background grown under 6h light/18h dark cycling or **C)** constant light conditions. Shown are mean values (+/- SEM) of biological replicates (n = 3). **D)** Representative images of maximum quantum yield of PSII (F_v/F_m) measured from three-day-old seedlings grown in the indicated light regiment.

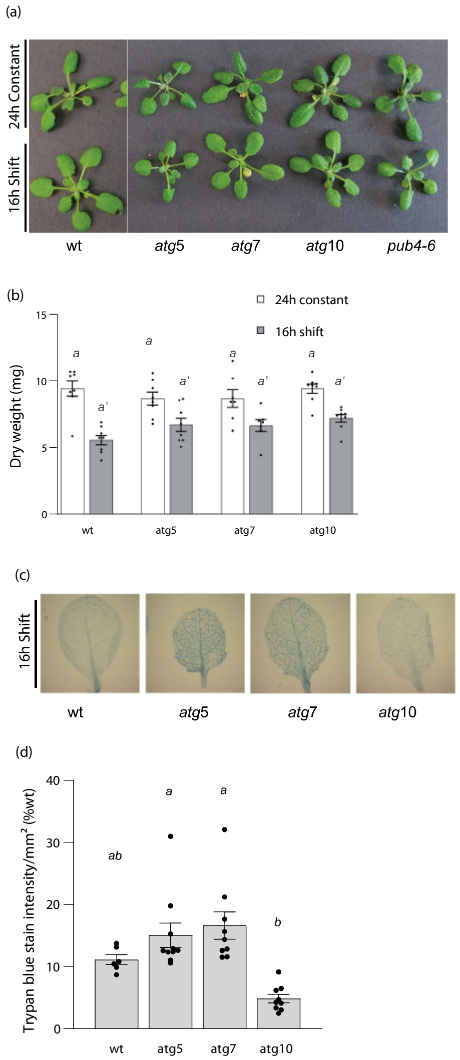

<u>Figure S5. Phenotypes of *atg5*, *atg7*, and *atg10* single mutant adult plants.</u>

The phenotypes of *atg* single mutant plants was assessed in the adult stage. **A)** Three-week old plants grown in 24h constant light or under stressed conditions (two weeks in 24h constant light and one week in 16h light/8h dark cycling light conditions). **B)** Mean biomass (+/- SD) of same plants (n = 8 plants). **C)** Representative trypan blue stains of single leaves from same plants. Lack of dark blue color is indicative of alive and healthy cells. **D)** Quantification of mean trypan blue signal (+/- SEM) in panel C ($n \geq 6$ leaves from individual plants). Statistical analyses were performed by one-way AVOVA tests followed by Tukey's HSD. Different letters above bars indicate significant differences (p value ≤ 0.05). For panel B, separate statistical analyses were performed for the different light treatments and the significance for the light stressed group is denoted by letters with a ′. In all bar graphs, closed circles represent individual data points.

Table S3. Mutant lines used in this study

mutant	Gene/locus	Mutation	Effect of mutation	reference
fc2-1	*FC2/* AT2G30390	GABI_766H08 T-DNA in 5'UTR	Reduced expression of *FC2* transcript	[3]
pub4-6	*PUB4/* AT2G23140	c9847535t	G225R	[1]
toc33	*TOC33/PPI1/* AT1G02280	c449839t	splice change, 3' end 2nd intron	[1]
atg5-1	*ATG5/* AT5G17290	SAIL_129_B07 T-DNA intron 4 of 8	Loss of transcript	[4]
atg7-2	*ATG7/* AT5G45900	GABI_655B06 T-DNA exon 7 of 11	Loss of transcript	[5]
atg10-1	*ATG10/* AT3G07525	SALK_084434 T-DNA exon 5 of 6	Loss of transcript	[6]

Table S4. Primers used in study

Gene	Oligo	Sequence
**Genotyping Primers**		
Salk LB	JP31 / LBb1.3	ATTTTGCCGATTTCGGAAC
Sail LB	JP848 / LB	GCCTTTTCAGAAATGGATAAATAGCCTTGCTTCC
GABI-KAT LB	JP286 / LB 08409	ATATTGACCATCATACTCATTGC
GABI-KAT RB	JP285 / RB 03144	GTGGATTGATGTGATATCTCC
fc2-1 GABI_766H08	LP JP283	GAGCAACGCCAAACATAGAAG
	RP JP284	TCAAAGGCAATGAATGTTTCC
atg5-1 SAIL_129_B07	LP JP1172	ATTTGCTATTTGTTTGGCACG
	RP JP1173	TACCGTTCATGACAGAGGTCC
atg7-2 GABI_655B06	LP JP1168	CGTGTAACAGTGCATTGTTGG
	RP JP1169	GGAGCTTAACAAAGGGAAACG
atg10-1 SALK_084434	LP JP1170	ACATAACCAATCGTTCCCTCC
	RP JP1171	ATCCTAAGACCAACCACCTGC
**qPCR primers**		
ACTIN2 AT3G18780	For JP199	GCACTTGCACCAAGCAGCAT
	Rev JP200	CCTTTCAGGTGGTGCAACGAC
**Stress Response Markers**		

SIB1 *AT3G56710*	For JP589	CAACCGGAGCCCATCTATT
	Rev JP590	GGAGAAAGGTTGTGGTCGTC
HSP26.5 *AT1G52560*	For JP585	CGAGCTTATCGTTGCCTGAT
	Rev JP586	CTCCGCCTTAATGTCCTCAA
BAP1 *AT3G61190*	For JP338	ATTGATGGATACGGTGGCCG
	Rev JP339	CAGACCCCAAACCGGAACTC
ATPase *AT3G28580*	For JP336	GAAGATCGGAAAAGCGTGGAA
	Rev JP337	CCGGGTGGTCCAAACAAAAG
ZAT12 *AT5G59820*	For JP344	GCGTTGGTTACACGCGCTT
	Rev JP345	CTTCAACGTAGTCACCGTGGG
CYC8 *AT4G37370*	For JP1130	AATGGGCATTGTCGAACGTG
	Rev JP1131	TGGTCGAAATGCCAAACTCC
**Starvation Response Markers**		
C-starve		
DIN6 *AT3G47340*	For WLO1757	GAGTTCCACTTCTCGGTGCA
	Rev WLO1758	GTGAGGGAAGATATGCCCCG
WCOR413 *AT4G37220*	For WLO1759	AAGGGGTGAGTTTTTGGCCA
	Rev WLO1760	CCAAACCGGGTAAACGAGGA
DRM1 *AT1G28330*	For WLO1761	GGGATGATGTTGTGGCTGGA
	Rev WLO1762	TCACCGCTGTACAACCAGTC
DIN11 AT3G49620	For WLO1826	AAAACGTGGACGGTGATTGG
	Rev WLO1827	ACATGTCACCGATGTTGCAG
DIN10 AT5G20250	For WLO1828	TCGCACCGTTAAGTTGCATC
	Rev WLO1829	ATAAAACGCGTCCCAAGTGC
N-starve		
NRT2.1 AT1G08090	For WLO1838	TTTTTGCCTGGCGACGTTTG
	Rev WLO1839	ACACAATGGGCATGAGCAAC
NRT2.4 AT5G60770	For WLO1840	AATGGCCGATGGTTTTGGTG
	Rev WLO1841	TTGGCTTTGTGTTCGGTGTC
NRT2.5	For WLO1842	TTTTGCTGCTTCGTCTCCAC

AT1G12940		
	Rev WLO1843	ACGATACGAGCGAAAACAGC
Autophagy-related Genes		
BAG6<br>*AT2G46240*	For WLO1630	GGACCCGGAGAATGCTAGTG
	Rev WLO1631	TTGCACGACGTCTGATCTGT
WRKY33<br>*AT2G38470*	For WLO1632	CCAAACCGAGACTCGTCCAA
	Rev WLO1633	TGCACTACGATTCTCGGCTC
RAB7<br>*AT1G22740*	For WLO1634	AATTCTTGGAGACAGCGGGG
	Rev WLO1635	AACCTCCTCTTTGCTCAGGC
ATG1B<br>*AT3G53930*	For WLO1636	TCTCAAGAAGACGGGTTGCC
	Rev WLO1637	TCCAGAGACCCATGGGAAGT
CDC48<br>*AT5G03340*	For WLO1638	GGCTCGTCAATCTGCTCCTT
	Rev WLO1639	CTGCAGCGTCTGAGCAAAAG
Core Autophagy Genes		
ATG5<br>*AT5G17290*	For WLO1751	ACAAATCCGACGTCGCTTCT
	Rev WLO1752	CTCCGTCTCAGGCACAACTT
ATG7<br>*AT5G45900*	For WLO1753	TGCCTTCTTCTTGGAGCTGG
	Rev WLO1754	CAACCACGTCGTTGCAGAAG
ATG10<br>*AT3G07525*	For WLO1755	TCGAGAGGTCAGCGATGGTA
	Rev WLO1756	ATCCAGTCCTCAGTCCCACA
ATG3<br>*AT5G61500*	For WLO1787	GATGGTTGGCTGGCTACACA
	Rev WLO1788	GGTTCTACTCCACGCGACAT
ATG4a<br>*AT2G44140*	For WLO1789	TCGTTTCTGGCAGCGAAGAT
	Rev WLO1790	ATCGCTGTGTGGGTTTGAGT
ATG4b<br>*AT3G59950*	For WLO1791	CGTGTATTGGCCGCATTCAG
	Rev WLO1792	GTTCACTCTGTCAAGCCCGA
ATG8a<br>*AT4G21980*	For WLO1793	CCTCTCGAGGCAAGGATGAG
	Rev WLO1794	TCAAGCAACGGTAAGAGATCCA
ATG8f<br>*AT4G16520*	For WLO1795	GAAGAGAAGGGCAGAGGCTG
	Rev WLO1796	CCAAATGTGTTTTCTCCGCTGT

ATG12a - AT1G54210	For WLO1797	GGAGTCGTCGTCCCCGAG
	Rev WLO1798	TCATCAGGGTTTGGCGAGAA
ATG12b - AT3G13970	For WLO1799	GGCGACCGAATCTCCGAATT
	Rev WLO1800	CTGATTCATCCGGGTTTGGC
ATG2 - AT3G19190	For WLO1801	CAAAGGGCCGGGATACAACT
	Rev WLO1802	CAGGTGACGAGACCAGCTTT
Predicted Microautophagy-related Genes		
ELC AT3G12400	For WLO1850	TTCGGCTGATCAGTCATTGC
	Rev WLO1851	TGGTGAACATGCTGCACTTG
VPS25 AT4G19003	For WLO1852	AGCTTCAATGCTTGCATGCC
	Rev WLO1853	AGAGGAACCTTGTTCCAAGTGG
VPS20.1 AT5G63880	For WLO1854	AACCCAAAGACGCAAGCTTG
	Rev WLO1855	TCTTGCAGCTTGCTTTTCCG
NBR1 AT4G24690	For WLO1856	ACTGGCGCTCATTCAAAGAC
	Rev WLO1857	AAACACGACGAGGATGCTTG
VPS15 AT4G29380	For WLO1858	TGCGTCAATTGCTTCTGAGG
	Rev WLO1859	TCTTTCGCTGTTTCGCCATG
NPC1 AT1G07230	For WLO1860	TGAGCACGGTGGGTTTTATG
	Rev WLO1861	AACACCCAATCGGTCAAACC
VAM3 AT5G46860	For WLO1862	AGATTCGACACAAGCCGTTG
	Rev WLO1863	TGCAACCTTGTCTTGTGCAG

References

1. Woodson JD, Joens MS, Sinson AB, Gilkerson J, Salome PA, Weigel D, Fitzpatrick JA, Chory J. Ubiquitin facilitates a quality-control pathway that removes damaged chloroplasts. Science. 2015;350(6259):450-454.
2. Sieńko K, Poormassalehgoo A, Yamada K, Goto-Yamada S. Microautophagy in Plants: Consideration of Its Molecular Mechanism. Cells. 2020;4(9):887-898.
3. Woodson JD, Perez-Ruiz JM, Chory J. Heme synthesis by plastid ferrochelatase I regulates nuclear gene expression in plants. Curr Biol. 2011;21(10):897-903.

4. Thompson AR, Doelling JH, Suttangkakul A, Vierstra RD. Autophagic nutrient recycling in *Arabidopsis* directed by the ATG8 and ATG12 conjugation pathways. Plant Physiology. 2005;138(4):2097-2110.

5. Hofius D, Schultz-Larsen T, Joensen J, Tsitsigiannis DI, Petersen NH, Mattsson O, Jorgensen LB, Jones JD, Mundy J, Petersen M. Autophagic components contribute to hypersensitive cell death in *Arabidopsis*. Cell. 2009;137(4):773-783.

6. Phillips AR, Suttangkakul A, Vierstra RD. The ATG12-conjugating enzyme ATG10 Is essential for autophagic vesicle formation in *Arabidopsis thaliana*. Genetics. 2008;178(3):1339-1353.
